# Old age and the associated impairment of bones' adaptation to loading are associated with transcriptomic changes in cellular metabolism, cell-matrix interactions and the cell cycle

**DOI:** 10.1016/j.gene.2016.11.006

**Published:** 2017-01-30

**Authors:** Gabriel L. Galea, Lee B. Meakin, Marie A. Harris, Peter J. Delisser, Lance E. Lanyon, Stephen E. Harris, Joanna S. Price

**Affiliations:** a School of Veterinary Sciences, University of Bristol, Bristol, UK; b Department of Periodontics & Cellular and Structural Biology, University of Texas Health Science Centre, San Antonio, USA

**Keywords:** MAPK, mitogen-activated protein kinase, Actn2, actinin alpha 2, AMPK, adenosine monophosphate-activated protein kinase, Atf3, activating transcription factor 3, ATP, Adenosine triphosphate, b2MG, Beta-2-microglobulin, Bcl11a, B-cell CLL/lymphoma 11A, Bcl212, Bcl-2-like protein 2, BiNGO, A Biological Network Gene Ontology tool, BMD, bone mineral density, BMP, bone morphogenetic protein, Ccna2, cyclin A2, Ccnd3, cyclin D3, Cdc20, cell division cycle 20, Cdca3, cell division cycle associated 3, Cdk6, cyclin dependent kinase 6, CO_2_, Carbon dioxide, Dab2IP, disabled 2 interacting protein, DAVID, Database for Annotation, Visualization and Integrated Discovery, Des, desmin, Dkk1, dickkopf WNT signalling pathway inhibitor 1, E2F1, E2F transcription factor 1, ECM, Extracellular matrix, EGF, epidermal growth factor, EGR2, early growth response 2, Ezh2, enhancer of zeste 2 polycomb repressive complex 2 subunit, FDR, false discovery rate, Fgf7, fibroblast growth factor 7, Fgfr1, fibroblast growth factor receptor 1, Fzd7, frizzled class receptor 7, Gnas, GNAS complex locus, Grb10, growth factor receptor bound protein 10, Gtse, G2 and S-phase expressed 1, Igf1, insulin like growth factor 1, Igf2, insulin like growth factor 2, Insig2, insulin induced gene 2, Itgb1, integrin subunit beta 1, Kcnma1, potassium calcium-activated channel subfamily M alpha 1, KEGG, Kyoto Encyclopaedia of Genes and Genomes, Mapt, microtubule associated protein tau, Mark3, microtubule affinity regulating kinase 3, MCM, Minichromosome maintenance protein complex, Mef2c, myocyte enhancer factor 2C, MEPE, matrix extracellular phosphoglycoprotein, Mki67, marker of proliferation Ki-67, MMP13, matrix metallopeptidase 13, Mmp25, matrix metallopeptidase 25, Myf6, myogenic factor 6, NADH, Nicotinamide adenine dinucleotide, Nusap1, nucleolar and spindle associated protein 1, PASTAA, Predicted Association of Transcription factors from Annotated Affinities, PBS, Phosphate buffered saline, PdgfA, platelet derived growth factor, alpha, Phex, phosphate regulating endopeptidase homolog, X-linked, Pitx2, paired-like homeodomain transcription factor 2, Pkia, protein kinase (cAMP-dependent, catalytic) inhibitor alpha, qRT-PCR, quantitative real time polymerase chain reaction, QTC, Quality Threshold Clustering, RarB, retinoic acid receptor beta, RNA, Ribonucleic acid, Scyl1, SCY1 like pseudokinase 1, Sost, sclerostin, Sox6, SRY-box 6, Sox9, SRY-box 9, Sp7, Sp7 transcription factor 7, SPEED, Signalling Pathway Enrichment using Experimental Datasets, Tcf15, transcription factor 15, TGFb, transforming growth factor beta 1, TNFa, tumor necrosis factor a, Ttn, titin, Vcam1, vascular cell adhesion molecule 1, VegfA, vascular endothelial growth factor A, Wnt16, Wnt family member 16, Ageing, Bone, Mechanical loading, Proliferation

## Abstract

In old animals, bone's ability to adapt its mass and architecture to functional load-bearing requirements is diminished, resulting in bone loss characteristic of osteoporosis. Here we investigate transcriptomic changes associated with this impaired adaptive response. Young adult (19-week-old) and aged (19-month-old) female mice were subjected to unilateral axial tibial loading and their cortical shells harvested for microarray analysis between 1 h and 24 h following loading (36 mice per age group, 6 mice per loading group at 6 time points). In non-loaded aged bones, down-regulated genes are enriched for MAPK, Wnt and cell cycle components, including E2F1. E2F1 is the transcription factor most closely associated with genes down-regulated by ageing and is down-regulated at the protein level in osteocytes. Genes up-regulated in aged bone are enriched for carbohydrate metabolism, TNFα and TGFβ superfamily components. Loading stimulates rapid and sustained transcriptional responses in both age groups. However, genes related to proliferation are predominantly up-regulated in the young and down-regulated in the aged following loading, whereas those implicated in bioenergetics are down-regulated in the young and up-regulated in the aged. Networks of inter-related transcription factors regulated by E2F1 are loading-responsive in both age groups. Loading regulates genes involved in similar signalling cascades in both age groups, but these responses are more sustained in the young than aged. From this we conclude that cells in aged bone retain the capability to sense and transduce loading-related stimuli, but their ability to translate acute responses into functionally relevant outcomes is diminished.

## 1 Introduction

The mass and structural organisation of bone tissue necessary to sustain functional loads without damage is established and maintained throughout life by the processes of bone modelling and remodelling. Bone formation by osteoblasts is coordinated with bone resorption by osteoclasts to ensure that at each location of each bone there is sufficient tissue appropriately aligned to withstand the mechanical loads to which it is subjected during physical activity. As the skeleton ages, an imbalance between bone formation and resorption occurs resulting in net bone loss. In humans, the extent of this loss can be sufficient that fragility fractures occur with minimal trauma. This is the major characteristic of post-menopausal and age-related osteoporosis. Similar age-related deterioration in bone structure occurs in mice; 19-month-old mice have dramatic reductions in cortical and trabecular bone relative to 19-week-old mice (Meakin et al., 2014, Galea et al., 2015a).

For bone formation and resorption to maintain appropriate bone mass and architecture, osteoblasts and osteoclasts must receive a functionally-relevant stimulus that they must follow with an adequate response. It is thought that the primary local stimulus for (re)modelling originates from the response of osteoblasts and osteocytes to the mechanical strains engendered by functional loading within the bone matrix. This stimulus interacts with systemic influences to orchestrate the activity of osteoblasts and osteoclasts in order to locally maintain bone's functional integrity. Mechanisms that could contribute to the age-related imbalance between bone formation and resorption include impaired osteocyte function and/or impaired osteoblast responses to “osteogenic cues”, including those resulting from mechanical simulation (Meakin et al., 2014). Mechanical loading triggers many acute responses, including the transcriptional regulation of hundreds of genes (Zaman et al., 2009). The functional outcome of any changes in gene expression depends not only upon their nature, but also the context in which they operate. For example, global depletion of the estrogen receptor ERα greatly diminishes the number of genes transcriptionally regulated following loading (Zaman et al., 2009).

It remains unclear whether the diminished adaptive response observed in old bones is a reflection of the acute responses of osteoblasts and osteocytes to loading being impaired or because the osteogenic context in which these responses occur has changed. We have recently demonstrated that several acute responses of osteoblastic lineage cells to mechanical strain are not significantly different between young and aged mice, including down-regulation of the Wnt antagonist sclerostin in osteocytes, up-regulation of the transcription factor Egr2, and entry of osteoblastic cells into the cell cycle (Meakin et al., 2014). If old age has little effect on the initial responses to strain this would suggest that age–related deterioration in the effectiveness of (re)modelling is due to reduced functional responses to the stimulus that strain engenders.

To begin to address this question, several in vitro studies have investigated replicative senescence transcriptomic changes as a model of ageing. Serial passage of human mesenchymal stem cells (osteoblast precursors) induces changes in genes related to proliferation, cell cycle progression and stress responses (Ryu et al., 2008). The bone marrow of 2-, 8- and 26-month old mice examined in vitro shows age-related changes in genes linked to differentiation, cell cycle progression and growth factors (Wilson et al., 2010). However, since ageing involves so many processes, in vitro models are severely limited. A previously-reported in vivo study examined the effect of ageing on gene expression in the bone of the cochlea and found that genes demonstrating age-related changes in expression were those involved in collagen maturation, extra-cellular matrix formation and bone mineralisation (Gong et al., 2006). A further microarray study compared gene expression patterns in transiliac bone biopsies from control and osteoporotic patients (Jemtland et al., 2011), reporting differences in the expression of the Wnt pathways antagonists (Sost, Dkk1) and bone matrix proteins (MEPE, MMP13). More recently, transcriptomic analysis of the response to loading in young versus old mice revealed blunted activation of canonical Wnt signalling, a potently osteogenic pathway, in the old following repeated bouts of loading (Holguin et al., 2016).

In this study we document age-associated differences in the basal transcriptome and in the in vivo loading-related transcriptomic responses of tibial cortical shells from young (19 week old) and aged (19 month old) female mice. Cellular processes and signalling pathways over-represented in the resulting list of differentially expressed genes were identified using a variety of bioinformatics techniques.

## 2 Materials and methods

### 2.1 Animals

19-week-old young adult and 19-month-old aged female C57BL/6 mice (36 of each age) were obtained from Charles River Inc. (Margate, UK). All mice were allowed free access to water and a maintenance diet containing 0.75% calcium (EURodent Diet 22%; PMI Nutrition International, LLC, Brentwood, MO, USA), 12-hour light/dark cycle, room temperature at 21 ± 2 °C. Cages contained wood shavings, bedding and a cardboard tube. Mice were housed in groups of up to 5 animals (Meakin et al., 2013). All procedures complied with the UK Animals (Scientific Procedures) Act 1986 and were reviewed and approved by the University of Bristol ethics committee (Bristol, UK).

### 2.2 In vivo external mechanical loading

Right tibias were subjected to one period of external mechanical loading under isoflurane-induced anesthesia. Left limbs were used as internal controls as previously validated (Sugiyama et al., 2010). The protocol for noninvasively loading the mouse tibia has been reported previously (Sugiyama et al., 2011, Sugiyama et al., 2012). The flexed knee and ankle joints are positioned in concave cups; the upper cup, containing the knee, is attached to an actuator arm of a loading device and the lower cup to a dynamic load cell. The tibia is held in place by a 0.5 N continuous static preload. Forty cycles of dynamic load are superimposed with 10 s rest intervals between each cycle. The protocol for one cycle consists of loading to the target peak load, hold for 0.05 s at the peak load, and unloading back to the 0.5 N preload. From the strain gage data previously reported (Meakin et al., 2014), different peak loads for young (15 N) and aged (12 N) mice were calculated to apply 2,500 με measured on the medial aspect of the tibia at the 37% site. Strain rate at this site was normalised to a maximum of 30,000 με s^− 1^.

### 2.3 RNA extraction and processing

**Supplementary Fig. 1:**
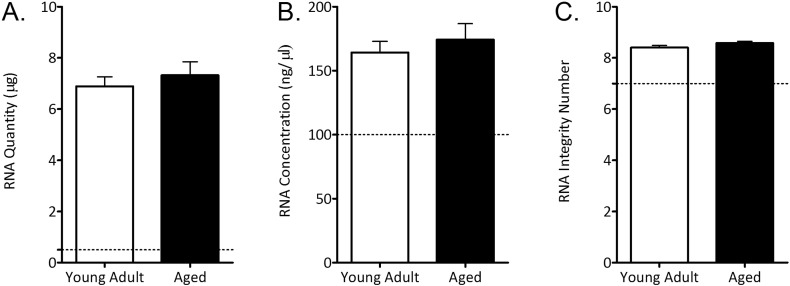
Quantity, concentration and integrity of RNA extracted from tibiae of young adult and aged female mouse tibiae. A) RNA quantity and concentration were calculated from NanoDrop spectrophotometry and B) integrity from Bioanalyzer electrophoresis. The dotted lines indicate the minimum recommended thresholds for microarray analysis (Meakin et al., 2014). Data represent mean ± SEM.

Mice were killed by CO_2_ asphyxiation and left tibiae immediately dissected from soft tissue attachments. The epiphyses were resected and marrow centrifuged out, leaving a cortical shell. Similarly-prepared samples predominantly contain osteocytes (Kelly et al., 2014). Shells were immediately snap frozen in liquid nitrogen. 2 tibiae in each group were pooled for RNA extraction giving 18 young and 18 aged bone samples. Bones in Qiazol lysis buffer were agitated with stainless steel ball bearings to disrupt the tissue according to the manufacturer's protocol (RNeasy Universal kit with TissueLyser LT, Qiagen). RNA was quantified using NanoDrop ND1000 and RNA quality assessed using a 2100 Bioanalyzer (Supplementary Fig. 1). RNA was converted to biotinylated hybridised cRNA using the Ambion Illumina TotalPrep RNA amplification kit following the manufacturer's instructions. Microarray analysis was performed at the Genomics and Microarray Core Facility at the University of Texas Southwestern Medical Centre at Dallas, USA. Microarray analysis was completed using Illumina MouseWG-6 v2.0 Expression BeadChip Kits, with probes for 45,281 probes that can measure over 21,000 coding gene expression levels in each sample. 6 chips were used for this experiment with 6 samples run on each chip.

### 2.4 Data normalisation

**Supplementary Fig. 2:**
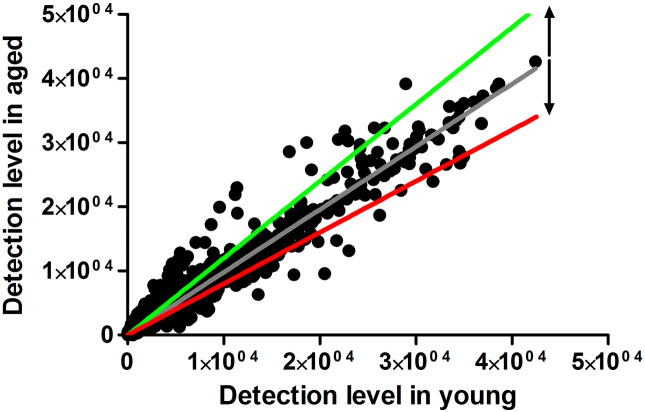
Gene expression values in tibiae from young mice are largely correlated to expression of the same genes in tibiae from aged mice. Following normalisation to a panel of housekeeping genes, average expression values for each gene across all samples from tibiae from young mice were compared with the corresponding values from tibiae from aged mice. There is a high overall correlation (R^2^ = 0.96, p < 0.001), indicating effective normalisation across age groups. The green line indicates a 20% increase in gene expression in the aged whereas the red line indicates a 20% decrease. Assuming the average microarray detection level and standard deviation, this study has an average power of 0.93 to detect 20% differences in expression at the 0.05 significance level.

GenomeStudio software (Gene expression module version 1.9, Illumina, San Diego, USA) was used to quantify gene expression in each sample. Quantile normalisation was used with background subtraction to correct gene expression levels in each sample against a panel of housekeeping genes. Corrected detection levels in tibiae from young mice were very closely correlated with those from aged mice (R^2^ = 0.96, p < 0.001, Supplementary Fig. 2). Loading responsive gene sets were determined by comparing detection values in loaded versus control limbs using Z tests at each time point, providing differentially expressed gene lists for each age groups at each time point. Benjamini corrections for multiple comparisons were then applied on each differentially expressed gene set.

### 2.5 qRT-PCR validation

Fold differences in the expression of differentially expressed genes, as quantified by microarray analysis, were compared against fold differences of the same genes in the same samples quantified by qRT-PCR. Equal quantities of RNA from each young and aged sample were collected to generate a pooled young sample and a pooled aged sample each containing 0.5 μg RNA and converted to cDNA using SuperScript II (Invitrogen) in a final reaction volume of 40 μl. 18 genes (1 μl cDNA per reaction with two technical repeats per gene leaving 4 μl pipetting error) were selected, including the housekeeping gene β2MG (primers previously described (Galea et al., 2014a)). Genes for validation were pre-selected to include both up-regulated and down-regulated genes related to proliferation (Bcl2l2, H1H2ag, Ccna2, Ccnd3, E2F1, H2H4, Igf1, Igf2), cell-ECM interactions (Itgb1, Des, Actn2, Ttn), transcription factors (Mef2c, Pitx2 and Myf6 in addition to proliferation and Wnt related transcription factors) and Wnt signalling (Tcf15, Wnt16). Primer sequences were from the Harvard Primer Bank (Wang et al., 2012) or designed using Primer-BLAST (NCBI) and are listed in Supplementary Table 1. qRT-PCR was performed as previously described (Galea et al., 2014a, Galea et al., 2014b).

To investigate whether differences in gene expression were specific for the tibia, femur cortical bone was collected from 8 additional young and aged mice. RNA was extracted, pooled and processed in the same way as the tibiae in order to quantify expression of the same 18 genes by qRT-PCR.

### 2.6 Bioinformatic analysis

#### 2.6.1 Gene ID conversion

Conversion of gene IDs between different platforms was performed using the Database for Annotation, Visualization and Integrated Discovery (DAVID) ID conversion tool (Huang et al., 2009).

#### 2.6.2 Functional cluster analysis

BiNGO analysis in Cytoscape (v3.1.2) was used to identify over-represented functional clusters in differentially expressed gene lists (Maere et al., 2005). Either the default or hierarchical layouts were used as appropriate. Where relevant, scalable vector graphic files of these clusters are provided as supplementary figures so that text can be magnified. Functional clusters enriched in the list of differentially expressed genes were also identified using DAVID (Huang et al., 2009). Only clusters in which all subgroupings were significantly enriched following Benjamini correction are reported.

#### 2.6.3 Enriched signalling pathways

Signalling Pathway Enrichment using Experimental Datasets (SPEED) analysis was performed to identify pathways over-represented in up- and down-regulated differentially expressed genes (Parikh et al., 2010). As the SPEED interface only accepts a maximum of 1000 gene IDs, those most up-regulated (out of 1422) and most down-regulated (out of 1515) were submitted as reported by others (Worton et al., 2013).

#### 2.6.4 Interlinked gene networks

To identify undirected protein-protein interaction networks, all differentially expressed genes were analysed in ReactomeFIViz (Wu et al., 2014) in Cytoscape v3.2.1 (Shannon et al., 2003).

#### 2.6.5 Enriched cellular processes

Kyoto Encyclopaedia of Genes and Genomes (KEGG) (Ogata et al.) analysis of cellular processes was performed on all genes differentially expressed between tibiae from young and aged mice through the DAVID interface. Significantly enriched processes manually annotated to indicate genes or clusters significantly up- or down-regulated in tibiae from aged versus young mice.

#### 2.6.6 Transcription factors associated with the ageing gene signature

Predicted Association of Transcription factors from Annotated Affinities (PASTAA) analysis (Roider et al., 2009) was performed to provide unbiased predictions of transcription factor affinity for the promoters of differentially expressed genes using default settings. The − 10,000 bp promoter regions conserved in mouse and human were considered. The entire list of differentially expressed genes was initially analysed, then down-regulated and up-regulated genes in tibiae from aged versus young mice were analysed separately.

To determine the number of genes potentially influenced by each transcription factor, TransFind analysis (Kiełbasa et al., 2010) was performed comparing the list of genes differentially expressed between tibiae of young and aged mice against a background list of non-differentially expressed genes (defined as microarray genes with p > 0.1) using the TransFac database (run parameters: high information, conserved in mouse and human, − 800 bp to + 200 bp promoters). This analysis is limited to 1 Kb promoters, unlike the much larger region analysed by PASTAA.

Transcription factor analysis was repeated using iRegulon (Janky et al., 2014). iRegulon was used to analyse loading-responsive gene signatures as it allowed temporal patterns to be investigated.

#### 2.6.7 Hierarchical clustering

Hierarchical clustering analysis was undertaken in MeV (Saeed et al., 2003) to determine whether the gene changes due to loading at different time points in the two age groups followed similar patterns. Unsupervised complete hierarchical clustering by Spearman's rank (categorical groups) was performed on the fold changes in gene expression between loaded and control limbs at each time point in both age groups.

#### 2.6.8 Quality Threshold Clustering (QTC)

QTC was performed in MeV (Saeed et al., 2003) to determine whether sets of loading-responsive genes followed similar temporal patterns of regulation. Loading-responsive genes in bones from young and aged mice were analysed separately. All genes significantly regulated by loading at any time point in each age group were analysed by Pearson's correlation (expression values).

### 2.7 Immunohistochemistry

Tibiae from 5 young and 5 aged mice were processed as previously described (Galea et al., 2014a). Haematoxylin and eosin staining was used to determine the proportion of empty lacunae as previously described (Meakin et al., 2014). Serial sections were collected from the tibial mid-shaft for immunohistochemistry. Primary antibodies (E2F1 H-137, p21 C-19) and non-immune negative control antibodies were from SantaCruz. Secondary antibodies were from Dako. Transverse tibial bone sections (6 μm) were stained using an indirect immunoperoxidase technique. Following deparaffinizing, antigen retrieval was performed using 10 mM citrate buffer solution (pH 6) (Sigma-Aldrich, Dorset, UK), at 95 °C for 30 min, then treated to inhibit endogenous peroxidase with 3% H_2_O_2_ in methanol (Sigma-Aldrich, Dorset, UK) for 30 min, washed three times in PBS and blocked for 1 h at room temperature in 10% donkey serum. Samples were incubated overnight at 4 °C in the primary antibody (1:100 dilution in 1.5% donkey serum in PBS) or equal concentrations of the non-immune negative control. The ImmunoCruz™ ABC Staining kit (Santa Cruz Biotechnology, Dallas, USA) was used for the remainder of the protocol following the manufacturer's instructions. Stained sections were imaged in the same region of the cortex under the same conditions on a Leica DMRB microscope with an Olympus DP72 digital camera. Images captured under 200 × magnification were exported to ImageJ (Schneider et al., 2012) and analysed blinded to primary antibody or age grouping. In ImageJ, oval regions were manually selected within each osteocyte lacuna and their intensity score obtained. Lacunae with scores < 115 were considered positively stained (empirically determined, smaller scores indicate greater staining). 57.2 ± 7 lacunae from 4 sections per bone were analysed.

### 2.8 Statistical analysis

Given the large sample size in the ageing comparison (n = 18 per group), assuming the average microarray detection level of 385 fluorescent units with an average standard deviation of 60, this study has an average power of 0.93 to detect 20% differences in expression at the 0.05 significance level (online calculator, University of British Columbia) due to ageing. Benjamini Hochberg correction was performed on p values calculated by Z tests using King's College London IoP's online correction tool (available at http://www.sdmproject.com/utilities/?show=FDR). Correlations were determined in GraphPad Prism or MeV. Comparisons between groups were done using Student's *t*-tests (two groups compared) or chi-squared tests (comparing proportions), p < 0.05 was considered significant. Enrichment scores and false discovery rates were calculated by the individual bioinformatics analysis tools through which they were generated.

## 3 Results

### 3.1 Ageing alters expression of genes involved in proliferation and bioenergetics related cellular processes

**Supplementary Fig. 3:**
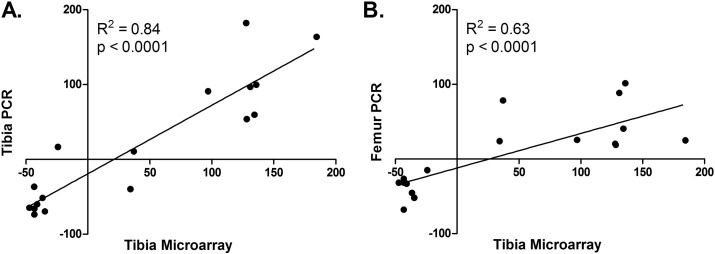
PCR validation of genes differentially expressed in aged versus young mouse bones. A) Equal quantities of RNA from all 18 samples from the tibiae of young mice and the equivalent samples from aged mice were pooled and the average expression of 18 pre-determined genes (including β2M as a house-keeping gene) was quantified in triplicate by qRT-PCR. Points represent the percentage difference in expression between the pooled sample from aged mice versus the pooled sample from young mice ([aged − young] / young ∗ 100) along the Y axis plotted against the percentage change for the same gene obtained by microarray analysis along the X axis. B) A similar approach to that in (A) was taken to compare the percentage difference in expression between a pooled sample from an independent group of 8 femurs for aged and young mice and microarray quantification of the same genes in the tibia.

2935 genes (19.8% of detected) were significantly differently expressed between the tibiae of young and aged mice. Of these, 1422 (48.4%) were up-regulated and 1515 (51.6%) were down-regulated in bones from aged relative to young mice (Supplementary Table 2). The fold differences in gene expression determined by microarray analysis were strongly and significantly correlated with fold differences quantified by qRT-PCR in the same samples (R^2^ = 0.84, Supplementary Fig. 3a) and in femurs of additional mice (R^2^ = 0.63, Supplementary Fig. 3b). 15 differentially expressed genes have previously been reported to contain polymorphisms associated with bone mineral density (BMD) in humans (Estrada et al., 2012): RarB (+ 167% in aged versus young), Pkia (+ 141%), Mapt (+ 99%), Kcnma1 (+ 45%), Sp7 (+ 39%), Mef2C (+ 37%), Scyl1 (+ 35%), Dab2IP (+ 32%), Grb10 (+ 26%), Insig2 (+ 25%), Mark3 (− 30%), Bcl11a (− 30%), Sox6 (− 42%), Sox9 (− 43%) and Wnt16 (− 43%). Wnt16 down-regulation was similar to our previous report of Wnt16 suppression in cortical bone of old male and female mice (Todd et al., 2015). Of the 138 genes currently (February 2016) associated with decreased bone mineral content by the International Mouse Phenotyping Consortium, 77 genes were differentially expressed between tibiae of young and aged mice (Supplementary Table 3, 56%, p < 0.0001 for this overlap occurring by chance given the proportion of detected transcripts in the array differentially expressed between age groups). These included genes with known functions in bone including Atf3 (+ 80.3% in the aged relative to young), Phex (+ 44.3%), Fgfr1 (+ 34.1%), Fgf7 (+ 26.6%), Gnas (+ 24.6%) and Ezh2 (− 38.6%).

**Supplementary Fig. 4:**
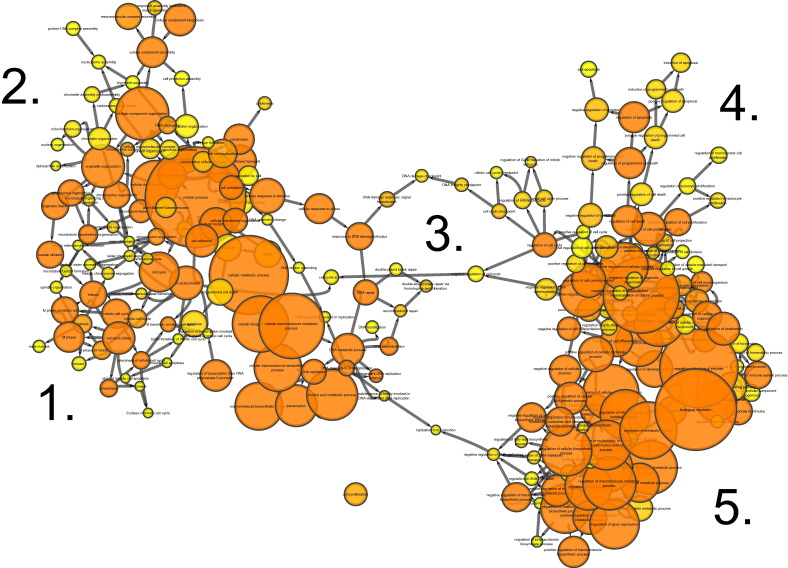
BiNGO interlinked networks of gene ontology terms related to proliferation which are enriched in the list of genes differentially expressed between young and aged tibiae.

**Supplementary Fig. 5:**
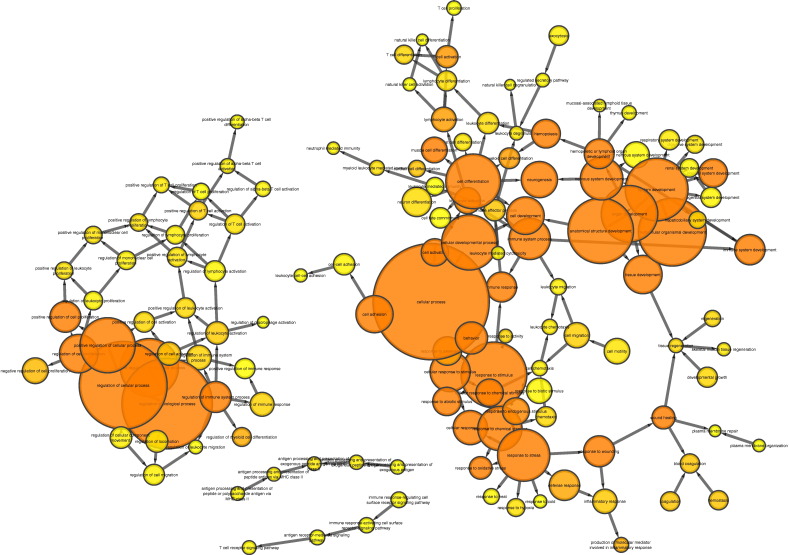
BiNGO interlinked networks of gene ontology terms related to inflammation which are enriched in the list of genes differentially expressed between young and aged tibiae.

**Supplementary Fig. 6:**
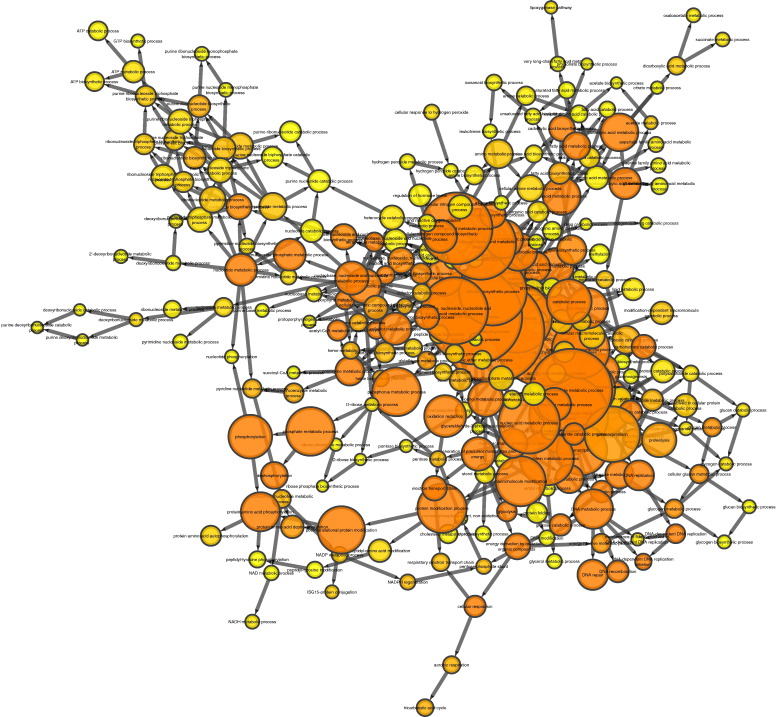
BiNGO interlinked networks of gene ontology terms related to bioenergetics which are enriched in the list of genes differentially expressed between young and aged tibiae.

**Fig. 1:**
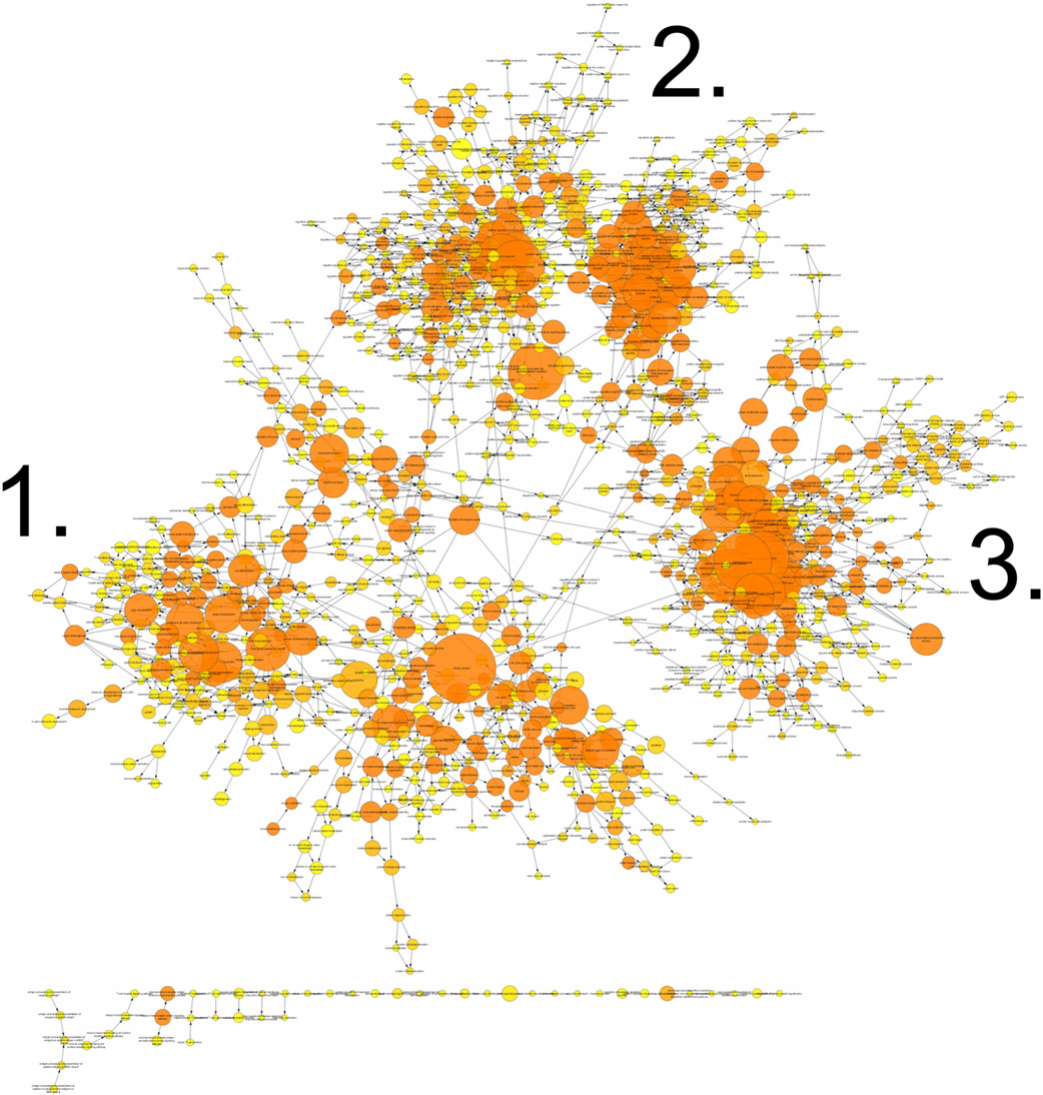
Enriched cellular processes in the list of genes differentially expressed between young and aged tibiae. BiNGO functional clustering analysis showing significantly-enriched clusters forming an inter-linked network clusters described in the Results.

BiNGO analysis of differentially expressed genes revealed networks of inter-related enriched functional categories which largely clustered into distinct groupings. These included clusters of enriched terms involved in (1) “organ development” including “skeletal development” and “skeletal system morphogenesis”, (2) cell cycle checkpoints and regulation of programmed cell death and (3) metabolic processes including “ATP biosynthetic processes” (Fig. 1). The proliferation-related signatures clustered in two groups; cluster terms related to cell cycle phases including M phase as well as chromatin assembly linked by the terms “DNA damage checkpoint”, “regulation of cell cycle” and “replication form protection” to a second cluster containing “apoptosis” and regulation of “metabolic process” terms (Supplementary Fig. 4). Proliferation also featured in an interlinked network primarily associated with inflammation (Supplementary Fig. 5). A large network of bioenergetics-related terms included both carbohydrate and lipid metabolism (Supplementary Fig. 6). DAVID analysis independently identified significantly enriched gene cluster groups related to mitochondria/energy metabolism (5 clusters) and the cell cycle (5 clusters).

### 3.2 Wnt, TGFβ and TNFα pathway components are altered with ageing

SPEED analysis revealed that genes significantly up-regulated in the tibiae of aged versus young mice are significantly enriched for components of the transforming growth factor (TGF)β (FDR-corrected p = 3.52 × 10^− 5^) and tumor necrosis factor (TNF)α (FDR-corrected p = 7.41 × 10^− 5^) signalling pathways. Genes identified by SPEED analysis as targets of the TNFα pathway included cytokines (Ccl5, 7, 8), a Wnt co-receptor (Fzd7), and transcription factors (Jun, Egr2 and Atf4). No TNFα ligands were differentially expressed. The TGFβ superfamily includes bone morphogenetic proteins (BMP) of which Bmp1 and Bmp8a were significantly up-regulated in bones from aged mice. TGFβ targets including various secreted signalling molecules (Fgf7, PdgfA and VegfA) and transcription factors (Jun, Egr2, vitamin D receptor).

In contrast, genes significantly down-regulated in the tibiae of aged versus young mice were enriched for components of the potently-osteogenic Wnt/β-catenin signalling pathway (FDR-corrected p = 0.0005), which promotes osteoblast proliferation and differentiation (Hartmann, 2006), and the mitogen-activated protein kinase (MAPK) cascade (FDR-corrected p = 0.032). MAPK-related genes included cyclin E, Cdk6, the transcription factor Sox9, the adhesion molecule Vcam1 and the matrix re-modelling enzyme Mmp25. Wnt pathway-related genes primarily included cell cycle regulators: Mki67, Cdca3, Gtse, Ctnpf, Nusap1, Cdc20. The Wnt ligand Wnt16 was significantly down-regulated, as we have previously reported (Todd et al., 2015), whereas Wnt10b was up-regulated in bones from aged mice.

**Supplementary Fig. 7:**
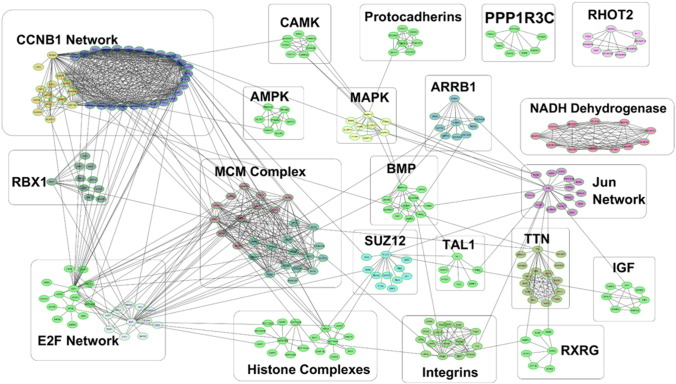
ReactomeFIViz clusters of 271 genes linked in networks are shown. Networks are manually annotated to indicate their predominant components (e.g. Histone complexes) or linking node gene (e.g. Rxrg).

**Supplementary Fig. 8:**
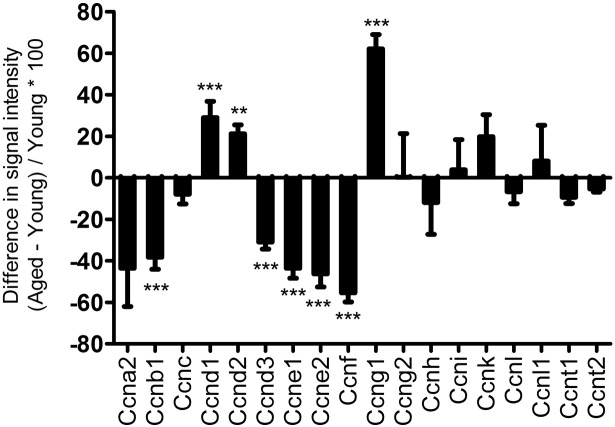
Microarray quantification of differences in cyclin expression between aged and young mice. Percentage difference in the expression of E2F factors detected in the microarray calculated as ([aged − young average] / young average ∗ 100). Bars represent the mean ± SEM percentage difference, n = 18, * p < 0.05, ** p < 0.01, *** p < 0.001.

**Fig. 2:**
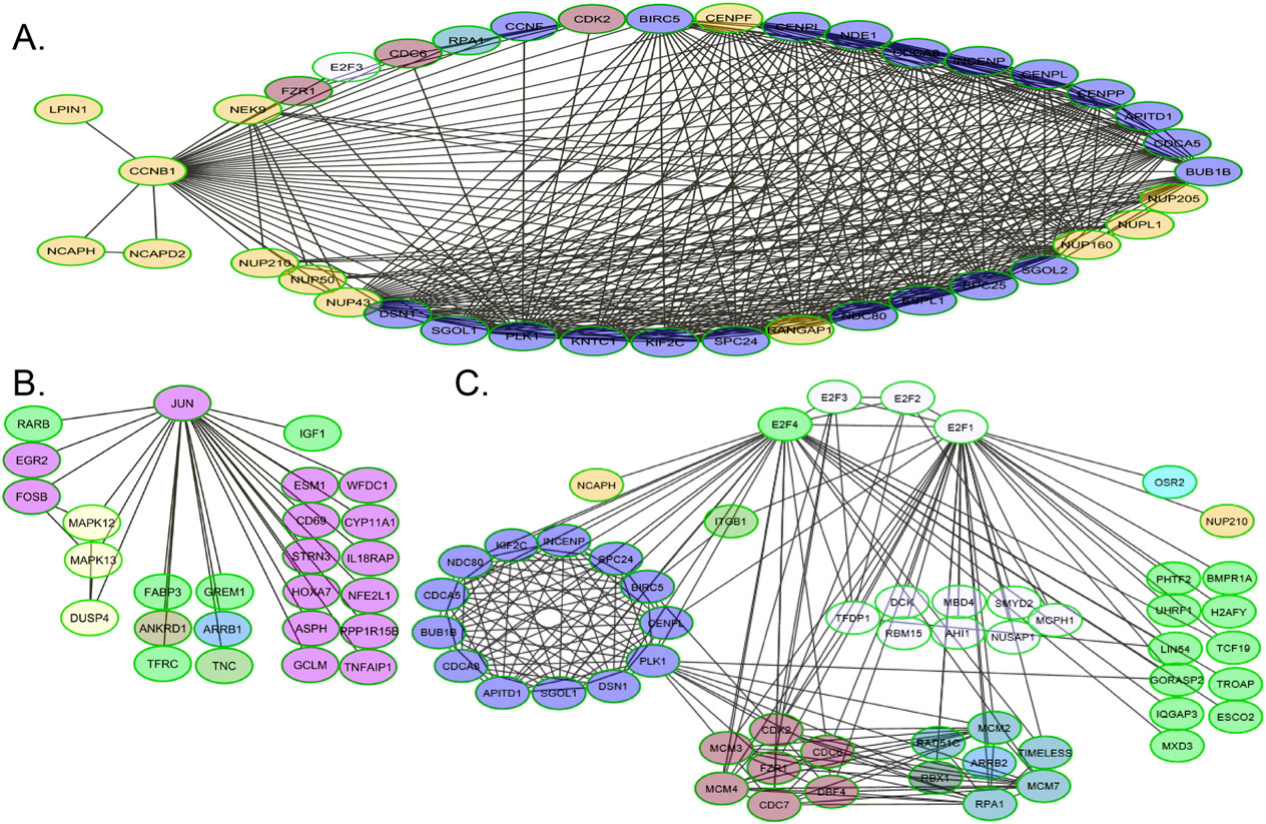
Interlinked gene networks identified in the list of genes differentially expressed between young and aged tibiae. ReactomeFIViz clusters of 271 genes linked in networks are shown. Immediate interaction partners of A) CCNB1, B) Jun and C) E2F1-4 identified in the list of differentially expressed genes by ReactomeFIViz are shown.

Wnt targets including Cdc20 (Hadjihannas et al., 2012) interact with the G2/M cyclin Ccnb1, which was identified as a central node linking a large number of differentially expressed genes (Fig. 2a and Supplementary Fig. 7a). The Jun transcription factor was also identified as a node interacting with various other transcriptional regulators (RarB, Egr2, FosB) as well as insulin-like growth factor (IGF) and MAPK pathways among others (Fig. 2b). Cyclins down-regulated in the aged include Ccnb1, Ccnd3, Ccne1-2 and Ccnf, whereas Ccnd1-2 and Ccng2 were up-regulated (Supplementary Fig. 8). The cyclin network interacts with the mini-chromosome maintenance (MCM) complex, proliferation-related networks involving E2F transcription factors and networks of genes coding for histone proteins. E2F genes formed a network linked to the MCM complex and various cell cycle regulators (Fig. 2c). Smaller networks related to integrins, protocadherins, IGF, MAPK, BMP, EGF, and other signalling molecules were also identified, as well as bioenergetics-related NADH dehydrogenase and AMP-activated protein kinase (AMPK) networks (Supplementary Fig. 7).

### 3.3 Ageing-associated transcriptomic changes involve cell-ECM interactions, carbohydrate metabolism and cellular proliferation pathways

**Fig. 3:**
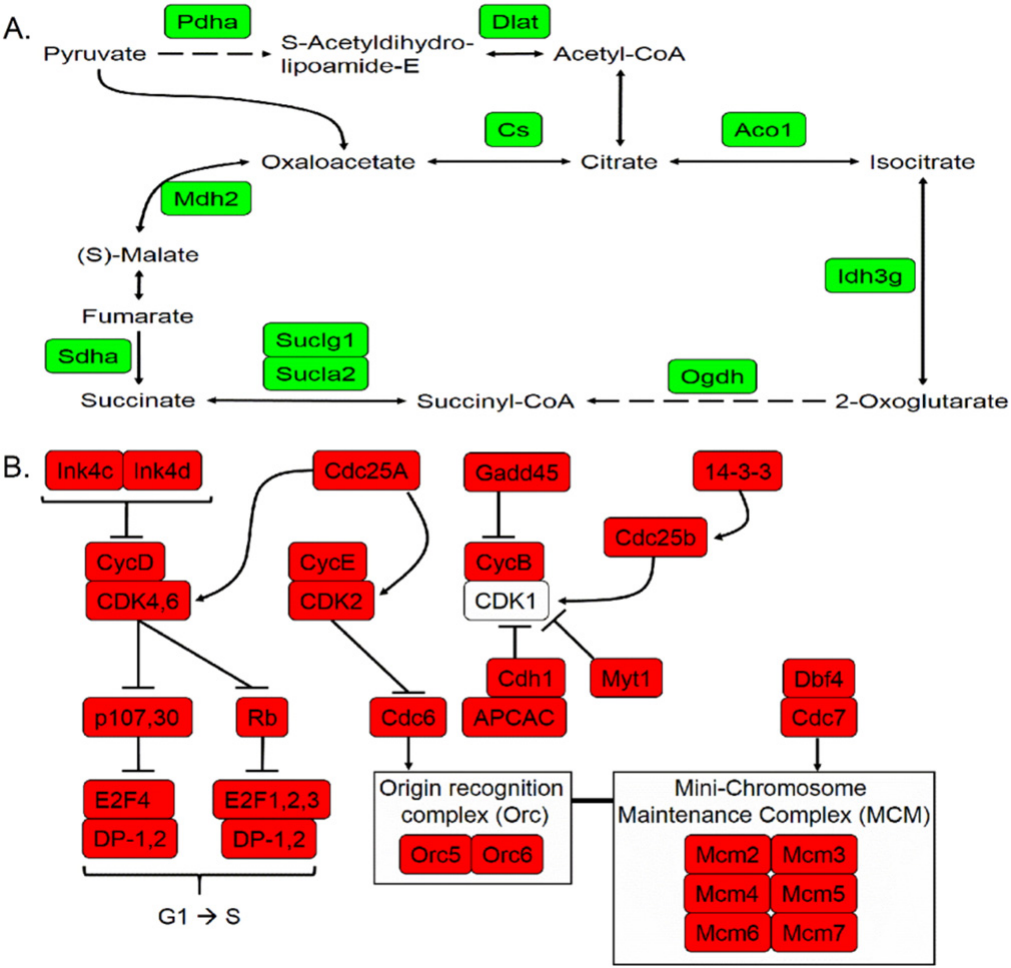
Differentially expressed genes between young and aged tibiae are significantly enriched for ECM-receptor interactions, TCA cycle and cell cycle pathway components. KEGG pathway analysis was performed on differentially expressed genes. Significantly enriched pathways related to A) ECM-receptor interactions linked to a “Hypertrophic Cardiomyopathy” (HCM) pathway, B) TCA cycle and C) the cell cycle. Green = up-regulated, red = down-regulated in tibiae from aged relative to young mice.

KEGG pathway analysis was undertaken to link differentially expressed genes into significantly enriched cellular processes. Components of the “Cell-extracellular matrix (ECM) interaction” KEGG pathway were significantly over-represented, including genes either up- or down-regulated in tibiae of aged versus young mice (not shown, includes integrin pathway components in Supplementary Fig. 7). A KEGG “Tri-carboxylic acid (TCA) cycle” pathway was also significantly enriched and remarkably all differentially expressed genes in this pathway were up-regulated in the tibiae of aged versus young mice (Fig. 3a). In contrast, genes involved in the significantly enriched “Cell cycle” KEGG pathway were down-regulated in the tibiae of aged mice (Fig. 3b).

**Fig. 4:**
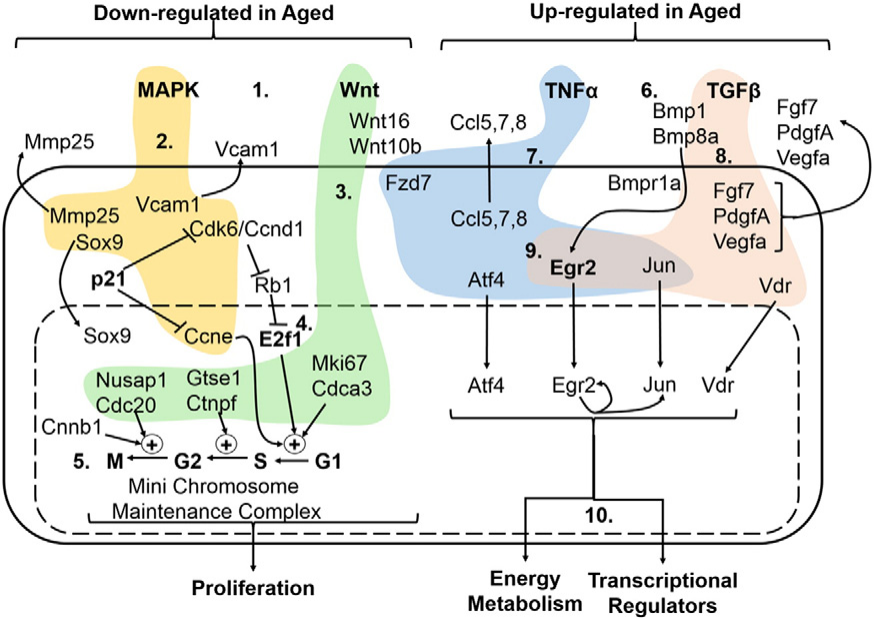
Schematic integration of genes, cellular processes and signalling pathways altered by ageing in the tibiae of mice. 1. Down-regulated genes in the bones of aged mice were enriched for components of the MAPK and WNT pathways. 2. MAPK targets included Mmp25, Vcam1, Sox9 and cell cycle regulators. 3. Wnt targets included cell cycle regulators of each stage of the cell cycle including the G2 ➔ M transition, in conjunction with the G2/M cyclin Cnnb1 identified as a central node. Wnt16 and Wnt10b were differentially expressed. 4. E2F1, which is down-regulated, was identified as a node linking pro-proliferative genes. 5. Components of the mini chromosome maintenance complex were also down-regulated. 6. Up-regulated genes were enriched for TGFβ and TNFα components. 7. TNFα targets include chemokines as well as transcription factors. 8. Egr2 and Jun are also targets of TGFβ/BMP signalling, which regulates various secreted signalling molecules. 9. Egr2 is the transcription factor with the highest affinity for the promoters of differentially expressed genes and interacts with Jun. 10. Up-regulated processes also include energy metabolism.

Combining BiNGO, SPEED and KEGG analyses suggests that genes differentially expressed in the young versus aged generally occurred in clusters that were either up- or down-regulated as a group. For example, genes involved in the tri-carboxylic acid cycle, TGFβ and TNFα pathway components were up-regulated in tibiae from aged mice, whereas cell-cycle related genes and Wnt pathway components were predominantly down-regulated (Fig. 4).

### 3.4 Potential involvement of transcriptional regulators in bone's ageing-related gene signature

We next investigated potential transcriptional regulators of these functional clusters. PASTAA analysis was used to identify transcription factors with the highest affinity for the promoters of differentially expressed genes. Egr2 had the highest overall affinity for differentially expressed genes and remained the transcription factor with the highest affinity when only genes up-regulated in the bones of aged mice were considered. The transcription factor most strongly associated with down-regulated genes was E2F1. TransFind analysis also revealed E2F1 and E2F2 are the transcription factors predicted to bind the greatest number of promoters of differentially expressed genes. Other over-represented transcription factors identified by TransFind included Sf1, Srebp and Mef2C, which were all themselves up-regulated in the microarray (not shown). iRegulon also identified E2F transcription factor responsive motifs as well as Mef2c targets as being over-represented in the genes differentially expressed between young and aged (not shown). Thus, three independent transcription factor analysis tools all identify E2F transcription factors typically associated with cell cycle regulation as being associated with the signature of genes significantly differently expressed between young and aged bone.

### 3.5 Ageing-associated E2F1 down-regulation in osteocytes

**Supplementary Fig. 9:**
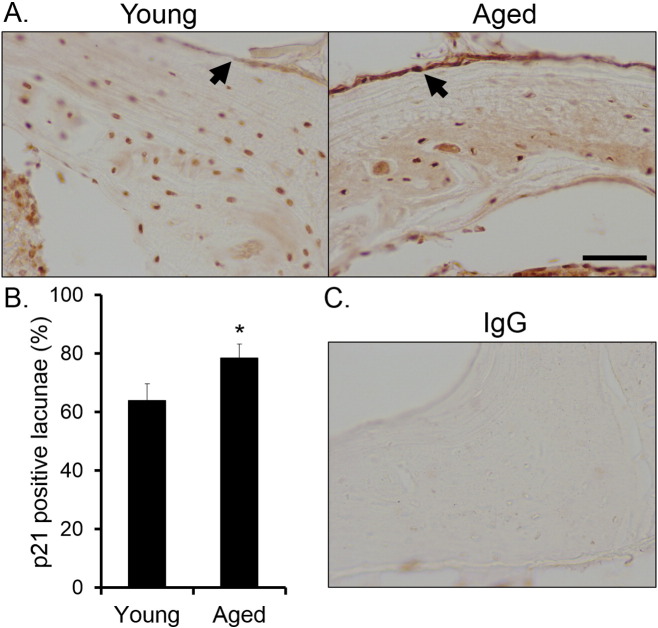
A greater proportion of osteocytes in tibiae from aged than young mice are positive for p21. A) Representative p21-stained tibiae mid-shaft cross sections from a young and an aged mouse. As well as positive staining in osteocyte lacunae, the periosteum (arrows) of sections from aged mice also subjectively appeared to stain more intensely (not quantified). Scale bar = 50 μm. B) Quantification of the proportion of positive lacunae corrected for the proportion of empty lacunae. C) Non-immune IgG negative control stained under the same conditions. Bars represent the mean ± SEM, n = 5. * p < 0.05.

Functional clustering analysis, pathway enrichment, gene network interactions and transcription factor analysis all indicate cell cycle components are a prominent feature of the gene signature in tibial cortical shells of old versus young mice. This is also consistent with the well-established age-related increase in osteocyte apoptosis (Jilka et al., 2007). Pro-apoptotic p21 was significantly up-regulated in the microarray (+ 38%), and the proportion of osteocyte lacunae positive for p21 was significantly greater in tibial mid-shaft cortical sections from aged than young mice (Supplementary Fig. 9).

**Fig. 5:**
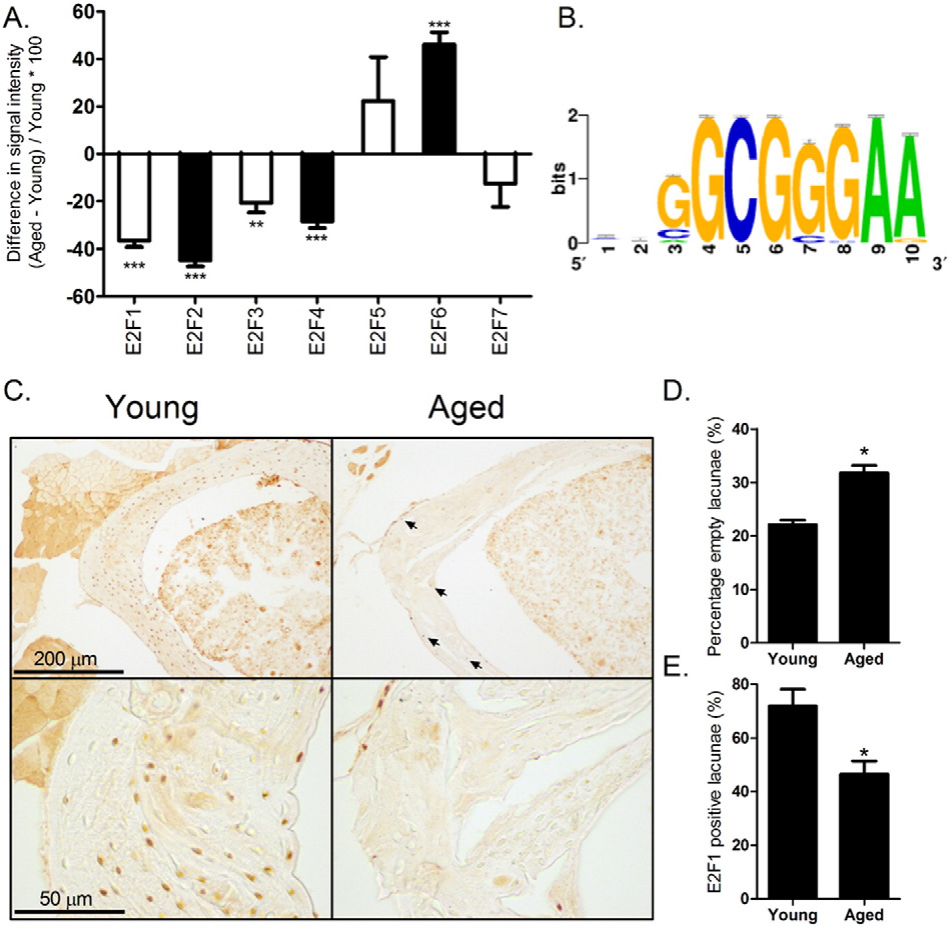
Ageing is associated with reduced E2F1 protein levels in the tibiae of mice. A) Percentage difference in the expression of E2F factors detected in the microarray calculated as ([aged − young average] / young average ∗ 100). B) E2F1 responsive motif predicted by iRegulon to be over-represented in the promoters of differentially expressed E2F1 target genes. C) Representative low and high power images of transverse cortical cross-sections taken through the mid-diaphysis of tibiae from young and aged mice immunohistochemically stained for E2F1. Arrows indicate some of the positive osteocyte lacunae seen at low power in the cortical bone of the aged mouse. D) Quantification of the proportion of osteocyte lacunae lacking a detectable cell body in H&E stained sections in transverse cortical cross-sections of tibiae from young and aged mice. E) Blinded, semi-automated quantification of the proportion of osteocyte lacunae stained positive for E2F1 following correction for the proportion of empty lacunae. Bars represent the mean ± SEM lacunae as a proportion of total lacunae, n = 5. * p < 0.05, ** p < 0.01, *** p < 0.001.

The pro-proliferative transcription factors E2F1-4 were all down-regulated in the bones of aged mice whereas E2F6 was up-regulated (Fig. 5a). A GGCGGGAA motif, was identified by iRegulon as being enriched in the promoters of differentially expressed E2F1 target genes (Fig. 5b). Osteocytes from young mice expressed E2F1 (Fig. 5c) and, despite accounting for empty osteocyte lacunae in sections from the same mice (Fig. 5d), the proportion of osteocyte lacunae stained positive for E2F1 was significantly lower in sections from aged than young mice (Fig. 5e). Thus E2F1, the transcription factor most strongly associated with the greatest number of down-regulated genes in the bones of aged mice, is itself down-regulated at the protein level in osteocytes, the predominant cell type present in cortical shells prepared as in the current study (Meakin et al., 2013).

### 3.6 Bones from both young and aged mice show extensive transcriptomic responses to loading

Consistent with the present finding that ageing alters the expression of proliferation and cell cycle-related factors, we have previously reported that cortical long bone osteoblasts derived from aged mice are less able to proliferate in response to mitogenic strain stimuli than similarly-derived cells from bones of young mice, due to an age-related disruption of cell cycle progression (Meakin et al., 2014). However, early responses to strain such as up-regulation of Egr2 were not significantly different between osteoblasts derived from bones of young and aged mice (Meakin et al., 2014). In the present study, loading regulated 4256 genes at any time point in young and 3355 in aged mice. 1383 genes were regulated by loading in both the young and the aged (p < 0.001 for this overlap occurring by chance compared with the proportion of total transcripts in the array significantly regulated by loading). The 1383 genes responsive to loading in both age groups include several with well-established functions in bone (e.g. Tnfrsf11a, Ctsk, Tcf4, Il11).

In addition to validating the microarray by comparison with qRT-PCR of the ageing signature described above, we also compared the loading responsive gene signatures from our study with a similar study in the ulnae of young rats reported by Mantila Roosa et al. (2011). Of the 1584 genes differentially expressed at 4, 12 or 24 h after loading in young rats which could be mapped to mouse equivalents, 425 genes were also regulated by loading in young mice in the current study (27%, p < 0.001 for this overlap occurring by chance, Supplementary Table 4). Similarly, 306 genes were regulated by loading in the aged mice in the current study and in the young rats in the Matilda-Roosa study (19%, p < 0.001, Supplementary Table 4). Overall, 145 genes were regulated by loading in the tibial cortical bone of both the young and aged mice in this study and in the ulnae of young rats in the Mantila-Roosa study (Supplementary Table 4), identifying a “mechanotranscriptome” of genes which are loading responsive across age groups, bones and species. The mechanotranscriptome includes known loading-responsive transcriptional regulators (including Egr1, Egr2), bone-specific genes (including Dmp1, Phex) and signalling molecules (including Igf2, VegfA).

### 3.7 Loading regulates many of the same functional categories and signalling pathways in bones from young and aged mice

**Supplementary Fig. 10:**
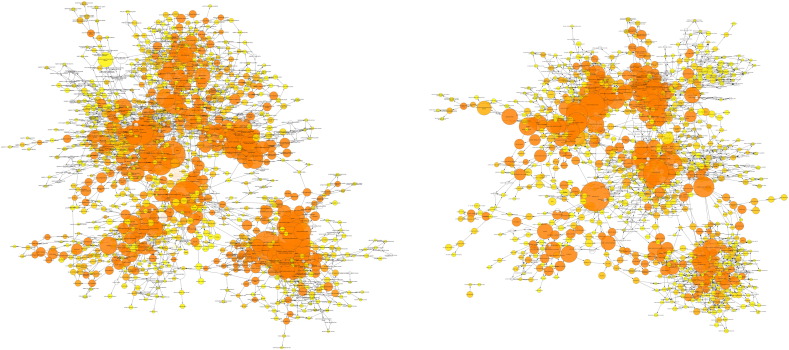
BiNGO interlinked network of gene ontology terms enriched in the lists of genes regulated by loading at any time point in either the young or aged mice.

**Fig. 6:**
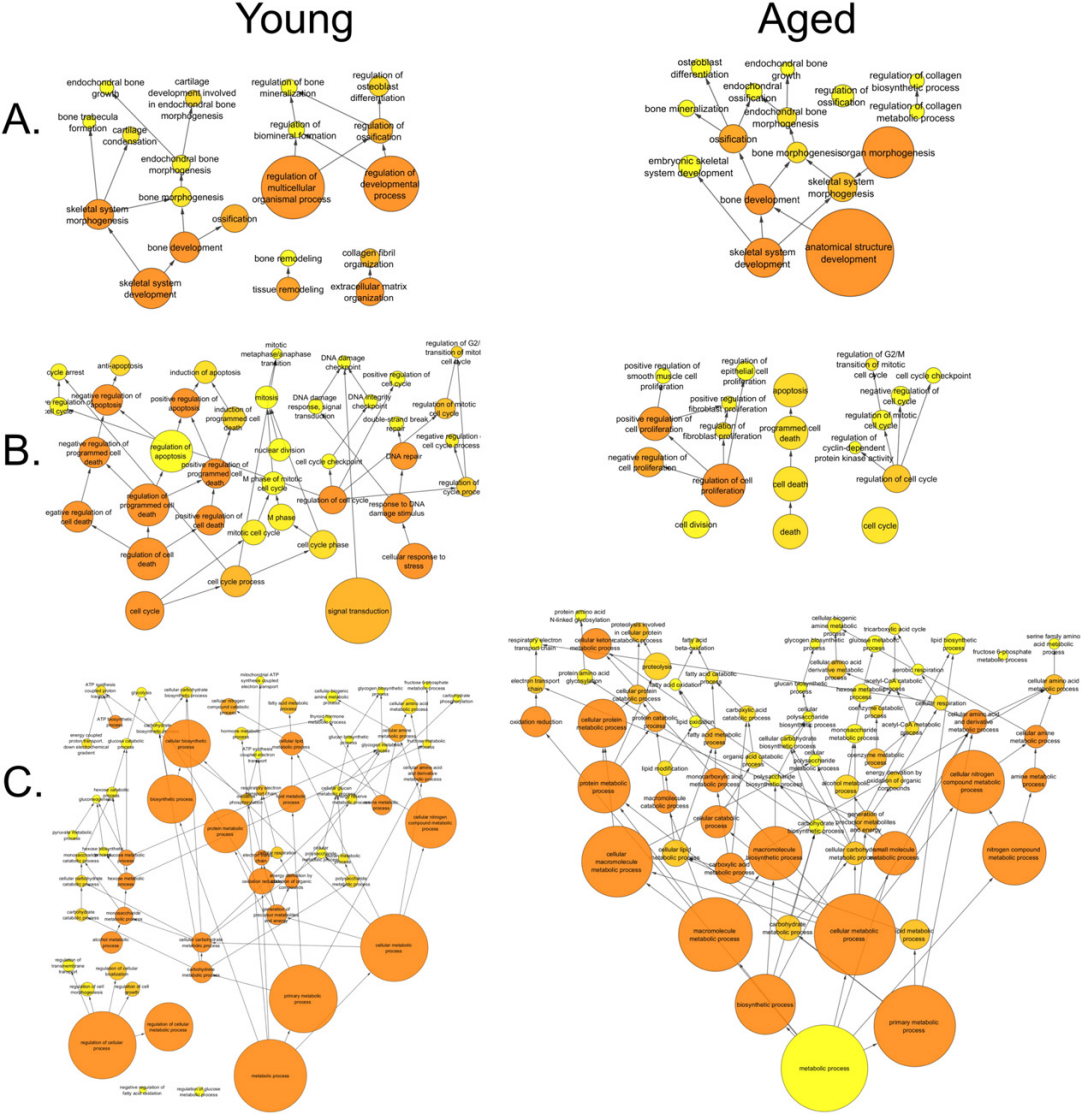
Loading-responsive genes are enriched for similar functional categories in both young and aged female mice. BiNGO analysis of all loading-responsive genes at all time points in each age group demonstrates inter-linked networks of enriched functional categories related to A) skeletal biology, B) the cell cycle and C) bioenergetics.

**Fig. 7:**
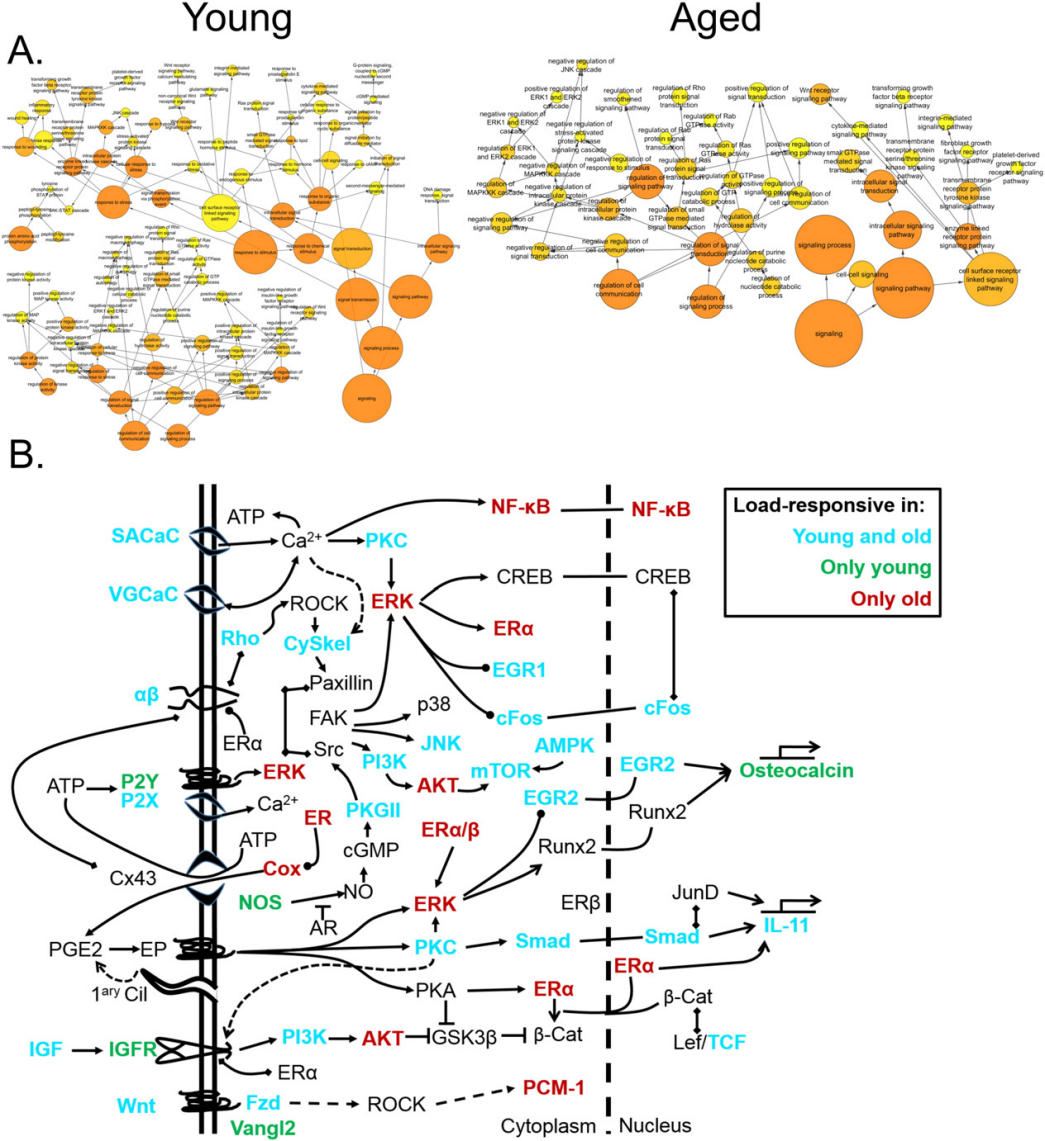
Loading regulates genes involved in known mechanoresponsive signalling cascades in both young and aged mice. A) BiNGO analysis of all loading-responsive genes at all time points in each age group identifying enriched functional categories associated with cell signalling. B) The mechanoresponsive cascades previously described in Galea et al. (2013) were compared against the lists of genes differentially expressed in the loaded versus control limbs of young and aged mice. Genes indicated in red were significantly up- and/or down-regulated at any time point following loading.

Analysing the overall loading-responsive gene signatures from young and aged mice using BiNGO reveals extensive interlinked networks of enriched functional categories, with the aged loading-responsive signature having a smaller overall number of categories, consistent with the signature containing fewer genes (Supplementary Fig. 10). Both the young and aged loading-responsive signatures were enriched for skeletal functional categories including “bone morphogenesis”, “ossification” and “osteoblast differentiation” (Fig. 6a). These categories contained bone-specific genes listed above. Both loading-responsive signatures contained categories associated with the “cell cycle” as well as “apoptosis”, although a greater absolute number of proliferation-related categories were enriched in the loading-responsive signature from young than aged mice (Fig. 6b). Both loading-responsive signatures were enriched for extensive networks of bioenergetics related processes including “cellular carbohydrate metabolic process” as well as “fatty acid metabolic process” (Fig. 6c). The loading-responsive signatures from both age groups were also enriched for “signalling”-related functional categories including Wnt, TGFβ, JNK and ERK1/2 MAPK pathways, PDGF, integrin and Rho cascades in both age groups. Prostaglandin and Jak-Stat signalling only featured in the young loading-responsive signature whereas smoothened and FGF only featured in the aged loading-responsive signature (Fig. 7a).

**Fig. 8:**
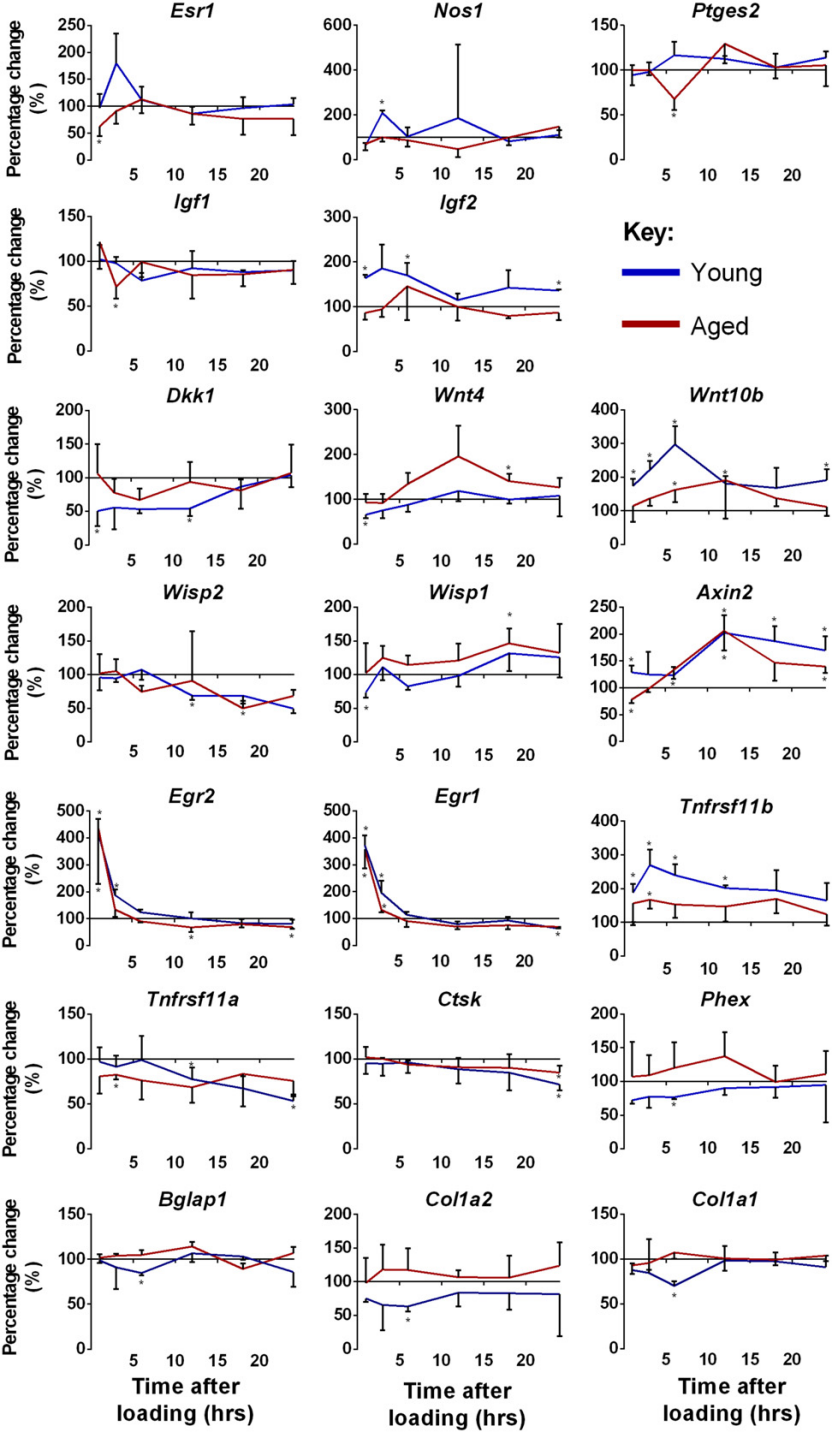
Loading regulated genes of key interest following a similar temporal pattern in young and aged mice, whereas others followed divergent patterns. The change in expression of key genes involved in mechanoresponsive pathways (Esr1, Nos1, Ptges2, Igf1, Igf2), Wnt pathway components (Dkk1, Wnt4, Wnt10b), Wnt target genes (Wisp2, Wisp1, Axin2, Egr2, Egr1, Tnfrsf11b) and re-modelling related genes (Tnfrsf11b, Tnfrsf11a, Ctsk, Phex, Bglap1, Col1a1, Col1a2). Points represent the mean percentage change ([loaded − control] / control ∗ 100) ± SEM, n = 3 in each age group at each time point. Blue = young, red = aged.

As no curated databases include bone-specific mechanoresponsive signalling pathways, we directly compared the genes regulated by loading at any time point against the list of mechanoresponsive pathway components we previously reported in Galea et al. (2013). Loading regulated components of calcium, integrin, estrogen receptor (ER), nitric oxide, prostaglandin (PG), Wnt and IGF signalling cascades in both young and aged mice (Fig. 7b). Individual genes in each pathway were identified and their loading-related changes in expression are shown (Fig. 8). Esr1 was down-regulated in the aged 1 h after loading, whereas it tended to be up-regulated in the young at 3 h. Nos1 was up-regulated in the bones from young but not aged mice at 1 h. Ptges2 tended to be up-regulated in the young but was significantly down-regulated in the aged at 6 h. Igf1 was down-regulated at 1 h only in the aged, whereas Igf2 was up-regulated only in the young at various time points.

Several canonical and non-canonical Wnt/Planar cell polarity pathway components were regulated by loading and both pathways are known to be mechanoresponsive (Galea et al., 2015b, Armstrong et al., 2007). The Wnt antagonist Dkk1 was down-regulated in the young but not aged at 1 h, the Wnt ligand Wnt4 was down-regulated in the young at 1 h and up-regulated in the aged at 18 h, whereas Wnt10b was up-regulated in the young at all time points except 18 h and was also up-regulated in the aged at 6 h. Various Wnt target genes were also regulated following loading and were similarly regulated in both age groups, although they did not follow a predictable pattern. Wisp2 tended to be down-regulated at late time points, Wisp1 and Axin2 both tended to be up-regulated at late time points, and Egr1-2 were up-regulated at early time points and down-regulated at late time points in both the young and aged.

Canonical Wnt signalling is known to regulate bone resorption in large part through the regulation of Tnfrsf11b (osteoprotegerin). Tnfrsf11b was up-regulated at 1–12 h in the young and at 3 h in the aged. Consistent with its up-regulation in both age groups, the osteoclast-associated markers Tnfrsf11a (RANK) and Ctsk (Cathepsin K) were down-regulated in both age groups at various time points. In contrast, Phex, Bglap1, Col1a1 and Col1a2 were down-regulated in the young but not aged at 3 h. Thus, various genes, including Wnt targets, follow similar patterns of change following a single episode of loading in both young and aged bones, whereas others such as those associated with bone formation show divergent patterns.

### 3.8 Age-specific regulation of loading-responsive processes

**Supplementary Fig. 11:**
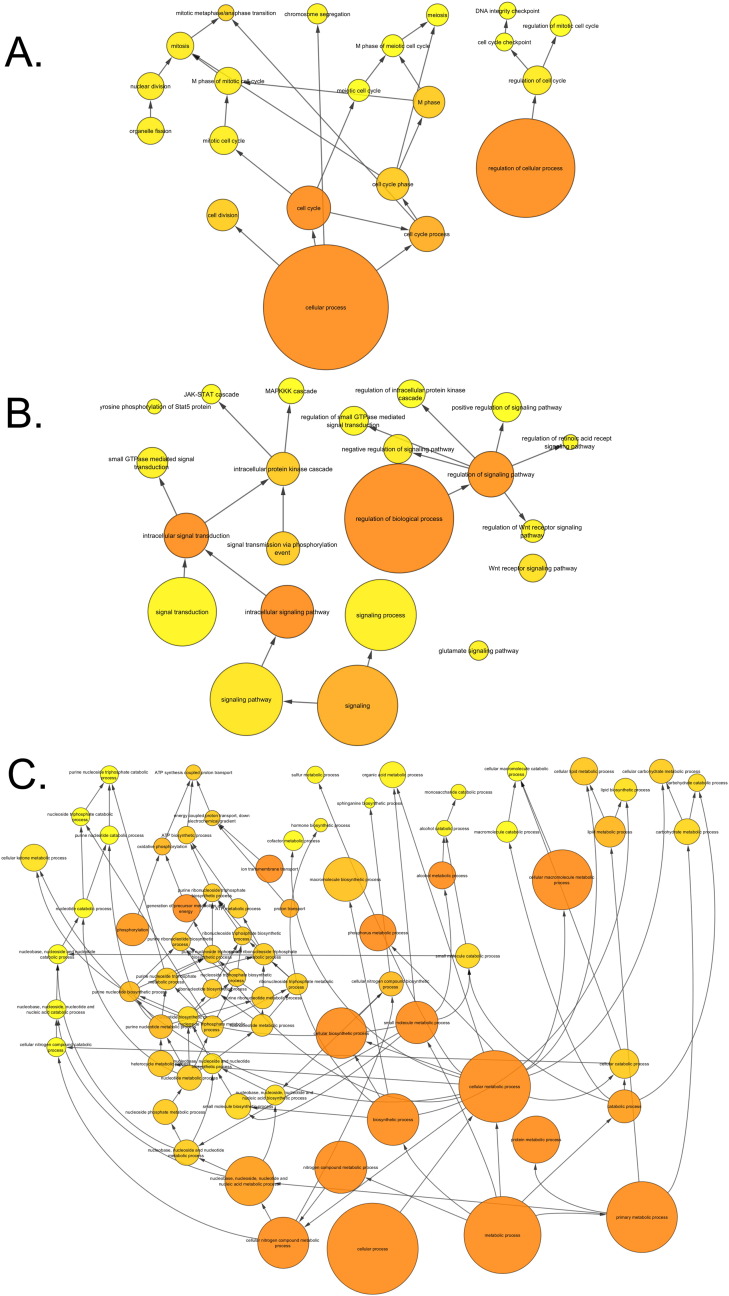
BiNGO interlinked networks of gene ontology terms which are only enriched in the genes regulated by loading in the tibiae of young mice. These networks are related to A) proliferation, B) signalling cascades and C) bioenergetics.

To begin to delineate differences between the transcriptomic responses seen in the two age groups, BiNGO analysis was performed on genes regulated by loading in one age group but not the other. When all time points were included, 3533 transcripts were regulated in the young but not aged mice following loading. Within this loading responsive signature observed in young mice, 1941 transcripts were up-regulated to a greater extent than they were down-regulated at any time point. Interlinked functional categories within this gene list include “cell cycle” categories particularly relating to “M phase” (Supplementary Fig. 11a) as well as signalling-associated categories such as “Wnt receptor signalling pathway” and “MAPKKK cascade” (Supplementary Fig. 11b). 1592 transcripts regulated by loading in the young but not aged mice were down-regulated to a greater extent than they were up-regulated at any time point. These were enriched for a large network of functional categories related to bioenergetics including “cellular carbohydrate metabolic process” and “ATP synthesis coupled proton transport” (Supplementary Fig. 11c).

**Supplementary Fig. 12:**
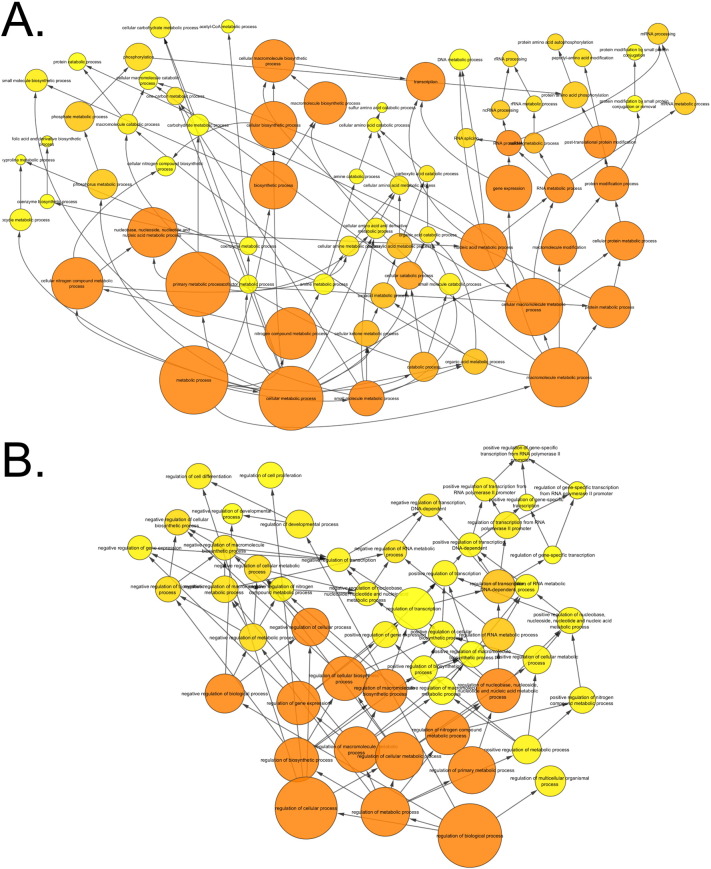
BiNGO interlinked networks of gene ontology terms which are only enriched in the genes regulated by loading in the tibiae of aged mice. These networks are related to A) bioenergetics and B) proliferation.

When all time points were considered, 2323 transcripts were regulated by loading in the aged but not young. Of these, 1338 transcripts were up-regulated to a greater extent than they were down-regulated at any time point. The signature of genes that was upregulated in old mice included functional categories relating to “transcription” interconnected to terms related to “cellular carbohydrate metabolic processes” (Supplementary Fig. 12a). In contrast, in young mice these processes predominated in down-regulated genes. The 985 transcripts down-regulated by loading in old mice but whose expression did not change in the young, were also enriched for “transcription” related terms, particularly “regulation of gene specific transcription”, as well as “regulation of cell differentiation” and “regulation of cell proliferation” terms (Supplementary Fig. 12b). Again this in contrast to the situation in young mice in which proliferation-related terms predominated in up-regulated genes.

### 3.9 Comparison of the effects of ageing and loading

**Fig. 9:**
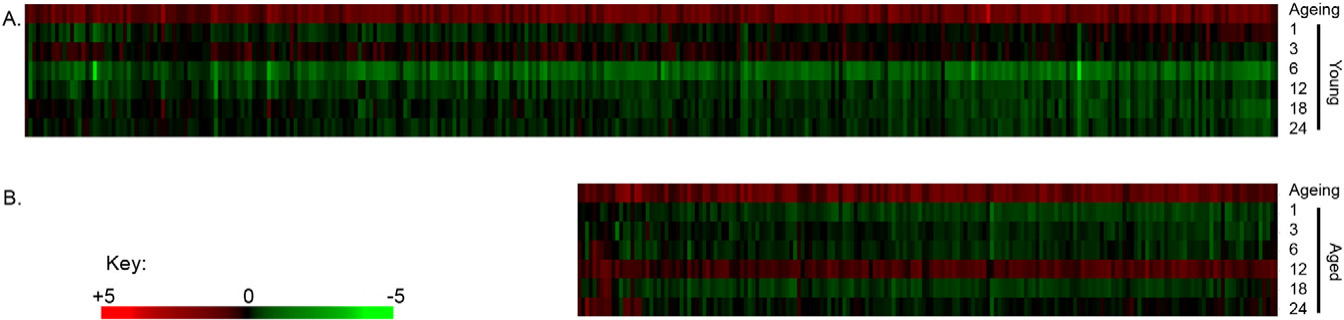
Bioenergetics-related genes up-regulated with ageing are down-regulated by loading in young mice but are up-regulated by loading in aged mice. Quality threshold clustering identified sets of genes which are up-regulated in the aged and which follow a similar temporal of change in expression following loading. A) In the young the cluster tends to be down-regulated 6 h after loading. B) In the aged the cluster tends to be up-regulated 12 h after loading. Both clusters are set up as follows (top to bottom): change in expression due to ageing followed by the change in expression 1, 3, 6, 12, 18 or 24 h after loading. Each column represents a different transcript. The key indicates log_2_ (fold change).

Given proliferation and bioenergetics related terms were enriched in the loading-responsive signature and featured prominently in the ageing-related signature, we also investigated whether loading “rescues” age-related transcriptomic changes. QTC clustering of the age-related gene signature and the young loading-responsive signature identified a large cluster of 331 transcripts which are up-regulated by ageing and down-regulated 6 h after loading in young mice (Fig. 9a): loading had the opposite effect to ageing on these genes. Genes in this cluster were primarily enriched for “generation of precursor metabolites and energy” related functional categories.

A similar comparison between the gene signature of old mice and their loading-responsive signature revealed a large QTC cluster of genes up-regulated in old bones and which were also predominantly up-regulated 12 h after loading (Fig. 9b). Remarkably, these genes were also primarily enriched for “generation of precursor metabolites and energy” related functional categories. This dichotomous pattern of loading-related regulation between age groups is consistent with the observation that load-responsive genes that are selectively down-regulated in young but up-regulated in old bones are enriched for bioenergetics-related terms.

### 3.10 Investigating the temporal pattern of gene regulation in the young and aged

**Supplementary Fig. 13:**
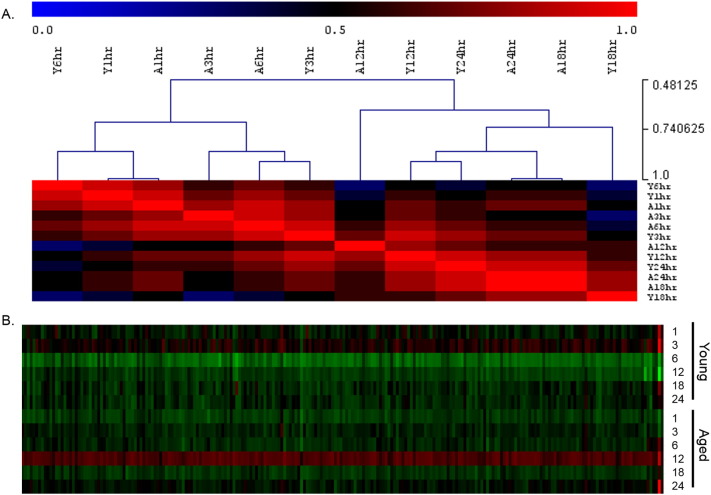
A) Non-negative matrix factorisation of all loading-responsive genes at all time points in both age groups showing the strength of correlation between changes in expression in different samples. B,C) Quality Threshold Clustering reveals a large cluster of genes which tend to be up-regulated at 3 h and down-regulated at 6 h following loading in the young, whereas in the aged these are predominantly up-regulated at 12 h. Panel B shows this data as a heat map whereas panel C shows the fold change with loading of each gene in the signature (grey lines) and on average (pink line) at each time point in young and aged mice.

**Supplementary Fig. 14:**
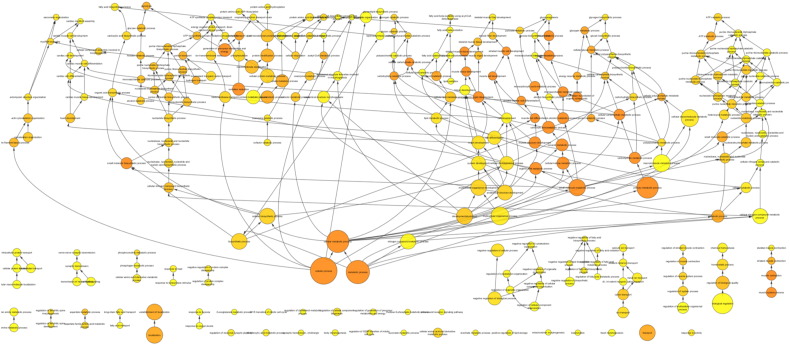
BiNGO interlinked networks of gene ontology terms which are enriched in the largest quality threshold cluster of genes dichotomously regulated by loading in the tibiae of young versus aged mice.

The analyses we have undertaken of multiple time points following loading provides a more comprehensive analysis of loading responsive biological processes than can be obtained from “snapshots” at any individual time point. The inclusion of different time points also allows temporal patterns of gene expression to be interrogated. Non-negative matrix factorisation of loading-related changes in expression of all transcripts regulated by loading at any time point in either age group revealed two primary clusters: one containing the early time points (1–6 h) from both young and aged mice and the second containing the later time points (12–24 h) from both age groups (Supplementary Fig. 13a). These temporal patterns were further investigated using QTC clustering. The largest QTC cluster contained 453 loading-responsive transcripts which were preferentially down-regulated 6 h after loading in the young and up-regulated 12 h after loading in the aged (Supplementary Fig. 13b,c). As in previous analyses, this dichotomous cluster was primarily enriched for bioenergetics related terms linked to “cellular metabolic processes” (Supplementary Fig. 14).

**Fig. 10:**
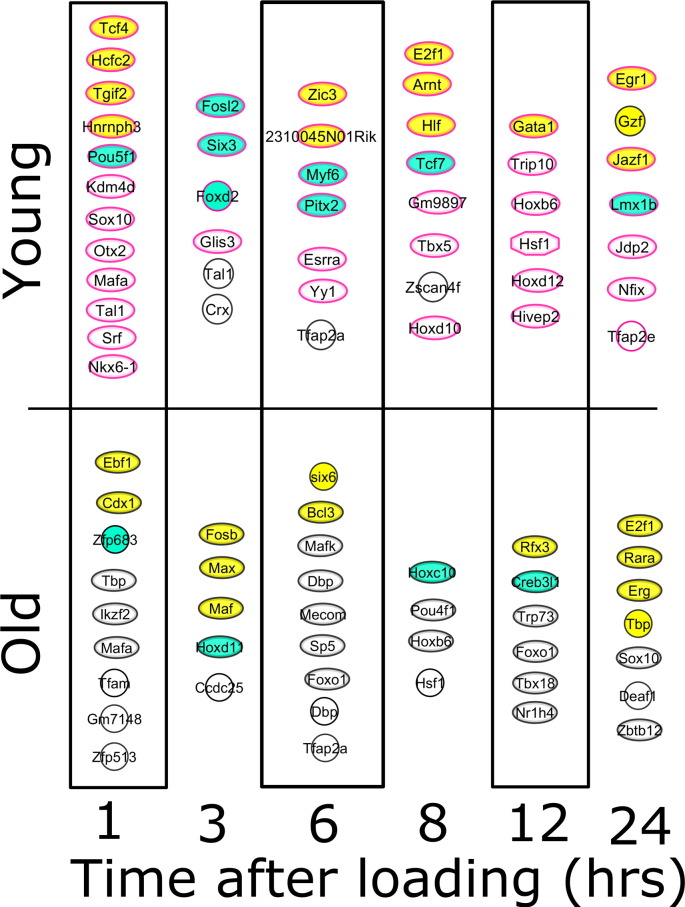
E2F1 is a predicted integral regulator of loading-responsive transcription factor networks in both young and aged tibiae. List of transcription factors whose targets are identified by iRegulon to be over-represented in the loading-responsive gene sets at each time point in each age group. Those indicated in cyan were themselves regulated by loading at the same time point at which their targets were enriched and those indicated in yellow were regulated by loading at other time points tested.

Dichotomous temporal regulation of co-expressed or co-repressed genes may reflect age-related differences in transcription factor activity. iRegulon analysis was therefore applied to the loading-responsive gene sets from each age group at each time point in order to identify transcription factors whose targets are over-represented. The targets of 46 transcription factors were over-represented in the loading-responsive signatures from young mice, of which 21 transcription factors were themselves regulated by loading (45.6%, p < 0.001 for this overlap occurring due to chance, Fig. 10). The targets of 44 transcription factors were over-represented in the loading-responsive signatures from aged mice, of which 19 transcription factors were themselves regulated by loading (43.2%, p < 0.001 for this overlap occurring due to chance, Fig. 10). Despite the large number of differences observed between the transcription factor networks identified in the two age group, there are also notable similarities. Mafa targets were over-represented in the genes regulated 1 h following loading in both age groups and both also included Pou, Hox and Six family transcription factors. E2f1 targets were over-represented in both age groups at late time points. Some of the over-represented transcription factors suggest involvement of specific cascades, such as Bcl3 in the aged bones indicating apoptosis-related signalling and Tcf7 as well as Tcf4 in the young bones suggesting involvement of the canonical Wnt signalling pathway.

**Fig. 11:**
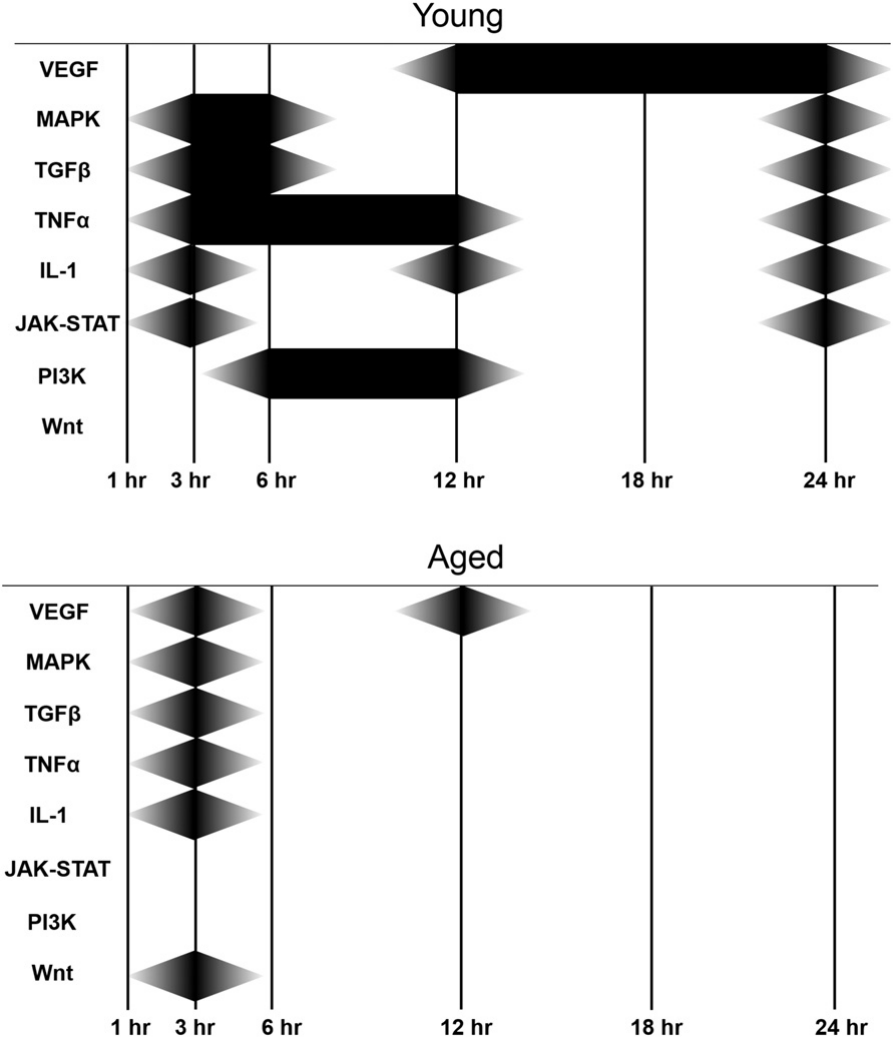
Temporal map of signalling pathways enriched in the loading-responsive gene sets in each age group. Schematic representation of signalling pathways identified as being over-represented by SPEED analysis of genes differentially expressed between control and loaded tibiae in each age group at each time point. Black diamonds indicate the time points at which each pathway was found to be over-represented in each age group.

To obtain a temporal map of loading-related changes in signalling pathways, SPEED analysis was applied to the genes regulated by loading at each time point in either young or aged mice. MAPK, TGFβ, TNFα and interleukin cascades were over-represented in the signatures from both age groups 3 h after loading, but the aged loading signature was also enriched for Vegf and Wnt pathway components whereas the young loading signature was also enriched for Jak-Stat at this time point. At later time points the temporal pattern of pathway activation diverged between young and aged, with the aged loading signature only showing enrichment at one other time point (Vegf at 12 h following loading). In contrast, the young loading signature showed sustained enrichment for Vegf between 12 and 24 h as well as enrichment for several pathways at 6, 12 and 24 h (Fig. 11).

## 4 Discussion

In this study we used a microarray approach to compare the basal transcriptome and the effect of axial loading in vivo on transcriptomic responses in the tibia of young adult and aged mice. Analysis of genes that were differentially expressed between the tibiae of young and aged female mice, validated by qRT-PCR and immunohistochemistry, identified a number of functional categories associated with cellular processes that were either significantly enhanced or diminished with age. Interestingly genes involved in a specific cellular process tended to be similarly up- or down-regulated; for example, proliferation-related genes were down-regulated in old bone. Acting on these different transcriptional backgrounds, mechanical loading resulted in rapid responses evident within one hour of loading in both age groups producing changes in gene expression which show significant overlap between the age groups and with those previously identified in young rats (Mantila Roosa et al., 2011) using similar methodology. Loading regulates many of the same cellular processes and signalling cascades in the bones of young and aged mice, however, the absolute number of regulated functional categories and the temporal pattern of pathway enrichment is diminished in the aged relative to the young. This indicates that bone cells of aged mice remain equally able to sense and transduce loading-related stimuli, but their ability to coordinate responses into functionally relevant outcomes is diminished.

We have previously compared bone morphometric parameters between similar mice from these two age groups, demonstrating a reduction in cortical area and thickness as well as reduced responsiveness to loading in the old mice (Meakin et al., 2014). The transcriptomic differences presented here should therefore be interpreted as a “snapshot” comparing an aged, low bone mass state to a young, structurally competent state. This comparison has identified differences in the expression of over half of all the genes found by the International Mouse Phenotyping Consortium to contribute to bone mineral content to date, as well as differences in the expression of at least fifteen genes that in humans contain polymorphisms associated with differences in BMD at the genome-wide level (Estrada et al., 2012). Differentially expressed genes also included bone matrix-related genes (Ibsp + 27% expression in aged versus young, Mmp25 − 40%), osteoblast transcriptional regulators (osterix + 39%, Sox6 − 41%, Vdr + 34%, Mef2c + 37%, Esrra + 33%, Ezh2 − 39%), and signalling molecules (Wnt16 − 41%, Lrp4 + 25%, Igf1 − 24%, Igf2 + 136%, Bmp1 + 34%, Bmpr1a + 79%) with known, although not exclusive, functions in bone. These differences illustrate the changed transcriptomic context in which any remodelling stimulus or therapeutic intervention attempting to increase bone mass must act.

Both old age and loading regulated functional clusters associated with proliferation/senescence and with bioenergetics. The expression of genes involved in inflammatory processes and mitochondrial function was also altered in aged mice. Age-associated low grade chronic inflammation, so-called “inflammaging” (Franceschi et al., 2007), is associated with cellular senescence and the generation of reactive oxygen species (ROS) by mitochondria during energy metabolism (Correia-Melo et al., 2016). It is increasingly recognised that not only is bone influenced by whole animal energy metabolism, but that changes in metabolic processes during osteoblast differentiation and function in turn influence remodelling (Riddle, 2015). For example, it has recently been reported that the osteogenic effects of parathyroid hormone (PTH) at least in part require a switch in osteoblast metabolism towards aerobic glycolysis down-stream of PTH-induced Igf signalling (Esen et al., 2015). In the present study Igf signalling pathway components were differentially expressed between bones from young and aged mice, as well as showing different patterns of regulation following loading; Igf2, but not Igf1, was significantly up-regulated by loading in young mice, whereas Igf1 was down-regulated in aged but not young mice. Dichotomous patterns of gene regulation following loading were also seen for other key mediators of bones' responses to loading including Esr1 (ERα) and Ptges2 (Cox2).

The most striking dichotomies between the two age groups were in the regulation of bioenergetics and proliferation related processes. Genes which function in proliferation-related categories tended to be up-regulated in the young but down-regulated in the aged following loading. This is consistent with rapid recruitment of bone cells to the cell cycle following mechanical loading and the rapid increase in the number of periosteal cells in the bones of young, but not aged mice, 24 h following loading (Meakin et al., 2014). Given that only small numbers of periosteal cells are present on the surface of the cortical shells used in our analysis and that the predominant cell type present is the terminally differentiated osteocyte (Kelly et al., 2014), the robust proliferation-related signature was unexpected. However, consistent with our observation, activation of Wnt signalling with anti-sclerostin antibodies has previously been reported to alter expression of proliferation-related genes in osteocyte-rich samples (Nioi et al., 2015, Taylor et al., 2016). One explanation for this proliferation-related signature is that it reflects changes in osteocyte senescence and predisposition to apoptosis, with apoptosis-related terms being significantly enriched in the loading-responsive gene signature from the aged mice as well as in the list of genes differentially expressed between young and aged. This explanation is consistent with the well-established increase in osteocyte apoptosis with ageing (Almeida, 2012), the reduction of osteocyte apoptosis induced by loading (Mosley et al., 2003), and the demonstration in the present study of increased p21-positive osteocyte lacunae in the tibiae of aged versus young mice.

The unexpected difference in the expression of cell cycle regulators in the tibiae of young and aged mice could be due to differences in their regulation or to cell populations being in different stages of the cell cycle. In the case of osteocytes, which do not proliferate, reduced expression of transcription factors conventionally associated with the cell cycle is likely to reflect differences in their regulation. We recently reported that mechanical strain, which induces proliferation in osteoblastic cells derived from young female mice, is able to recruit cells from aged female mice to the cell cycle, but those from aged mice showed delayed G2 ➔ M progression (Meakin et al., 2014). These findings are consistent with the down-regulation of cell cycle regulators in aged bones in the present study, and the identification of the G2/M phase cyclin Ccnb1 (− 40% in the aged), as a central node interacting with a large cluster of differentially expressed genes. Age-related reduction in Ccnb1 expression has previously been reported in human T lymphocytes (Quadri et al., 1998). Whether the down-regulation of Ccnb1 is a cause or consequence of the ageing-related dysregulation of cell cycle processes in bone remains to be determined, as do its functions in osteoblastic lineage cells.

Expression of the conventionally pro-proliferative transcription factor E2F1 was robustly detected in osteocytes from young mice, but its expression was significantly lower in aged bones. While the roles of E2F1 in osteoblastic lineage cells are poorly understood, its over-expression increases expression of alkaline phosphatase, collagen 1 a1 and osteocalcin (Yu et al., 2013), markers of differentiation rather than proliferation. In another study, E2F1 was found to directly bind the promoter of receptor activator of nuclear factor κB ligand (RANKL) (Hu et al., 2008). This transcriptional regulator therefore has the potential to influence osteoblastic cell functions beyond those related to proliferation. The genes predicted by TransFind to be directly regulated by E2F1 and E2F2 include several transcription factors (E2F1-3, Ccne1-2, FosB, Tcf19), nuclear and chromosomal structural proteins (Mcm2-6, Tipin, H2 sub-units) and epigenetic regulators (Ezh2 and Dnmt1). E2F1 transcriptional networks were also identified in the loading-responsive gene signature of both young and aged mice. Thus, basal differences in the expression of E2F1 may begin to explain basal as well as a loading-responsive transcriptomic differences in young and aged mice. However, the roles of E2F1 in loading responsive gene networks are largely unknown and need to be explored.

In assessing transcriptomic responses to loading we have previously identified Egr2 as the mechano-responsive transcription factor associated with more pathways and cellular functions than any other load-responsive gene (Zaman et al., 2012). In the present study Egr2 was basally up-regulated in the aged versus young. Although its target genes were not significantly enriched following loading in the current study, the closely-related transcription factor Egr1 was identified as a transcriptional node controlling loading-responsive genes in young, but not old, mice. Interestingly both Egr1 and Egr2 were two of the 145 genes similarly up-regulated by loading in the tibiae of both young and aged mice in the present study as well as in the ulnae of young rats as reported by Mantila Roosa et al. (2011). We suggest that this list represents a “mechanotranscriptome” of key loading-responsive genes. Beyond providing candidates which can be used as positive controls for the effects of loading, further interrogation of the function of genes in the “mechanotranscriptome” may help identify conserved mechanoreceptors and down-stream signalling mechanisms involved in their regulation. Many such cascades have already been described and a large proportion of the genes involved in these pathways were regulated by loading in the current study. This includes components of both canonical and non-canonical Wnt signalling and it is interesting that even though Wnt signalling genes were basally down-regulated in aged bones, several canonical Wnt target genes were regulated by loading and followed similar temporal patterns in both the young and aged.

Similar acute activation of Wnt signalling following a single episode of tibial loading has recently also been observed in a study by Holguin et al. (2016) who concluded that “old mice have a normal Wnt response after a single loading bout” based on analysis of sclerostin down-regulation and TOPGAL reporter activation. In the Holguin study (Holguin et al., 2016), transcriptional analysis of tibiae from young and aged mice following repeated bouts of loading showed that activation of the Wnt pathway is more transient in the old and whereas young mice repeatedly (or persistently) activated Wnt signalling following repeated bouts of loading, old mice did not. Detailed temporal analysis in the present study reinforces the conclusion that loading responses in the old are more transient than in the young, but this effect is unlikely to be limited to Wnt signalling, as indicated by more persistent activation of several of the loading responsive pathways in the SPEED database analysed in the young. The findings in the present study that a smaller overall number of genes are regulated by loading in the old than in the young and that bone-related genes such as Bglap1 and Col1a1 are suppressed at early time points selectively in the young, are also consistent with the findings of the Holguin study (Holguin et al., 2016). Differences between the studies, potentially due to differences in the loading protocols used and time points assayed, include differences in the Wnt ligands found to be regulated by loading. Holguin et al. (2016) did not observe changes in the canonical Wnt ligand Wnt10b within the first day following loading, whereas enhanced Wnt10b levels were observed acutely following loading in the current study, as well as in previous studies (Johnson et al., 2006, Kelly et al., 2016).

Old age has been associated with altered Wnt signalling in various tissue types as well as bone, in part because Wnt/β-catenin signalling can induce expression of telomerase responsible for extending telomeres (Hoffmeyer et al., 2012). In the present study, the expression of Wnt10b was higher in old bone (+ 34%), but the expression of Wnt16 was lower (− 41%), as we have previously reported (Todd et al., 2015). Osteoblastic cell-derived Wnt16 acts as a critical inhibitor of osteoclastic activity such that its deletion results in reduced bone mass and spontaneous fractures selectively in cortical bone in mice (Movérare-Skrtic et al., 2014). The anti-resorptive effects of Wnt signalling in bone include its ability to prevent bone loss caused by over-activation of TNF signalling (Yu et al., 2014). It is therefore noteworthy that the TNFα pathway was significantly enriched in the genes observed to be up-regulated in old bones, together with TGFβ. Age-associated up-regulation of TGFβ signalling has previously been reported in various cell types (Quéré et al., 2014, Yan et al., 2014) and, in the current study, two ligands of the BMP subfamily were up-regulated in the aged mice; BMP1 (+ 34%) and BMP8a (+ 28%). TGFβ signalling is most commonly associated with matrix production and remodelling (Tang and Alliston, 2013) and it is therefore intriguing to speculate that alterations in this pathway could explain some of the alterations in the significantly-enriched cell/ECM attachment pathway.

Alterations in cell/ECM attachments, particularly changes in the expression of components of the integrin pathway recognised to be an important mechano-receptor mechanism (Thi et al., 2013), could impair osteoblastic responses to strain. Integrins, together with Wnt, MAPK and other pathway components, were over-represented in the loading-responsive gene signature from both the young and aged. While these observed similarities in the loading signature between young and old animals reinforces the importance of some established pathways as core components of bones' responses to loading, differences should help identify why functional responses to loading are compromised in old bone. For example, it is intriguing that prostaglandin signalling-related functional categories were enriched in the loading-responsive signature from young, but not aged mice. Prostaglandin signalling is known to be mechano-responsive and facilitates bones' adaptation to functional loading, at least in part, through interactions with the canonical Wnt pathway (Galea et al., 2011).

Analysis of the temporal pattern of pathway over-representation following loading suggests that age-related deficiencies extend beyond individual cascades. Whereas loading-related transcripts in young mice clustered in coherent signalling cascades forming a sustained temporal pattern of pathway over-representation over the several time points tested, the loading responses in aged mice showed similar enrichment, but only at early time points after loading. This finding reinforces our hypothesis that cells in the bones of aged mice remain able to sense and respond to acute changes in their mechanical loading environment, but that their responses are hampered by deficiencies in the osteogenic context in which these stimuli act.

In summary, in the cortical bone of the tibia of female mice (that primarily contains osteocytes), old age is associated with both up-regulation and down-regulation of parts of the transcriptome. Genes that are up-regulated in aged bones are primarily associated with altered carbohydrate metabolic activity, TGFβ and TNFα signalling. Down-regulated genes in aged bones primarily involve components of the Wnt and MAPK pathways and expression of cell cycle-related transcriptional regulators including E2F1. The presence of E2F1 in osteocytes, which do not proliferate, suggests that it has poorly understood functions beyond the initiation of cell proliferation. The same fundamental cellular processes of proliferation and energy metabolism are involved in the early responses of bone cells to mechanical loading. This means that although cellular responses to loading in old mice are initiated from an altered transcriptomic baseline, bone cells in old mice remain equally capable of sensing loading-related stimuli and transducing these stimuli into rapid regulation of large numbers of genes. Many genes, including Egr1-2, follow similar temporal patterns of regulation by loading in both age groups whereas others, such as those related to proliferation and bioenergetics, show divergent patterns. The commonality in genes and processes affected by ageing and by loading, together with the observation that acute responses to loading are unaffected in old bones, suggests that age-related changes in the adaptive response are a consequence of changes in the osteogenic context in which loading acts. The genes and mechanisms underlying this “context” are poorly defined, but are likely to include those which determine the magnitude, onset, duration, localisation and coordination of acute responses to (re)modelling stimuli. This context must include the numbers of cells present to execute responses as well as the responsiveness of those cells to perceived stimuli. Therapies intended to prevent or reverse the ageing-related decline in skeletal functional competence should aim to rescue the cellular context in which such stimuli occur. Such therapies are likely to have far-reaching benefits beyond bone.

The following are the supplementary data related to this article.

Supplementary Table 1 Description of qRT-PCR primers used to validate the microarray. Supplementary Table 1

Supplementary Table 2 Complete list of gene target and Illumina probe IDs, mean and standard deviation (StDev) of the detection value in young and aged, the percentage difference in expression between young and aged ([Aged − Young] / Young), and the p value associated this difference. Supplementary Table 2

Supplementary Table 3 List of gene IDs of all genes differentially expressed between the tibiae of young and aged mice which are also associated with reduced bone mineral content in the International Mouse Phenotyping Consortium. Supplementary Table 3

Supplementary tables Image 1

## Funding

GLG and LBM were supported by Wellcome Veterinary Intercalated Training Fellowships (088560/Z/09/Z and 092045/Z/10/Z) under the supervision of LEL and JSP and GLG is currently funded by a Postdoctoral Clinical Training Fellowship also from the Wellcome Trust (107474/Z/15/Z). PJD was supported by University of Bristol internal funding under the supervision of LEL and PJS. MAH and SHE NIH R01DE024797.

## Disclosures

All authors state they have no conflict of interest.
