# Supplementary material for: Old age and the associated impairment of bones' adaptation to loading are associated with transcriptomic changes in cellular metabolism, cell-matrix interactions and the cell cycle
Source: Gene. 2017 Jan 30;599:36–52. doi: 10.1016/j.gene.2016.11.006 (PMC5139832; doi:10.1016/j.gene.2016.11.006)
Supplement: Supplementary Table 1 — Description of qRT-PCR primers used to validate the microarray. [file mmc1.docx]

**Supplementary Table 1:** Description of qRT-PCR primers used to validate the microarray.

| **Gene** | **PrimerBank ID** | **Amplicon Size** | **Forward Primer** | **Reverse Primer** |
| --- | --- | --- | --- | --- |
| **Des** | 33563249c2 | 106 | GTTTCAGACTTGACTCAGGCAG | TCTCGCAGGTGTAGGACTGG |
| **E2f1** | 158517881c1 | 97 | GAGAAGTCACGCTATGAAACCTC | CCCAGTTCAGGTCAACGACAC |
| **Bcl2l1** | 118129881c1 | 77 | ACATCCCAGCTTCACATAACCC | CCATCCCGAAAGAGTTCATTCAC |
| **Hist1h2ag** | (Primer-BLAST) | 70 | CTGCCCAAGAAGACCGAGAG | TCAACAGTGCTTTGTATAAAGGGT |
| **Wnt16** | 255683340c3 | 114 | AGTGCAGGCAACATGACCG | CCACATGCCGTACTGGACATC |
| **Ccnd3** | 126012503c1 | 132 | TGCGTGCAAAAGGAGATCAAG | GGACAGGTAGCGATCCAGGT |
| **Ttn** | 12844570a1 | 154 | GACACCACAAGGTGCAAAGTC | CCCACTGTTCTTGACCGTATCT |
| **Myf6** | 118130704c2 | 189 | CTGAAGCGTCGGACTGTGG | ATCCGCACCCTCAAGAATTTC |
| **Igf2** | 170172555c3 | 93 | CCGAGAGGGACGTGTCTAC | GTCTCCAGGTGTCATATTGGAAG |
| **TCF15** | (Primer-BLAST) | 152 | TGCTTGAAAGTGAGGGGTGT | CAGGAGTGGTTTGGGCTTCT |
| **Mef2c** | 281485602c1 | 113 | ACGAGGATAATGGATGAGCGT | ATCAGTGCAATCTCACAGTCG |
| **Pitx2** | 109948273c2 | 107 | ACCCCGGCTATTCGTACAAC | GGACAGGGGATTGACGTTCAT |
| **Itgb1** | 254910968c2 | 155 | ACTGTGATGCCGTATATTAGCAC | GATATGCGTTGCTGACCAACA |
| **CCNA2** | 161353443c1 | 118 | AAGAGAATGTCAACCCCGAAAAA | ACCCGTCGAGTCTTGAGCTT |
| **Hist2H4** | 21361209a1 | 67 | GGTGGAAAGGGTCTAGGCAAG | CCTGGATGTTGTCACGCAAGA |
| **IGF1** | 163659888c1 | 220 | CACATCATGTCGTCTTCACACC | GGAAGCAACACTCATCCACAATG |
| **Actn2** | 157951642c2 | 197 | ATGCGGTTCCACAAGATTGC | AGCCCTTCTTTGGCAGATGTT |
